# GSPT1-specific protein degradation is effective in preclinical models of chemoresistant *MYCN*-amplified neuroblastoma

**DOI:** 10.1186/s13046-026-03647-0

**Published:** 2026-02-06

**Authors:** Aleksandra Adamska, Hanna Chahin, Erick Andrés Muciño-Olmos, Javanshir Esfandyari, Kristina Aaltonen, Sofia Granados-Aparici, Joachim Tetteh Siaw, Katarzyna Radke, Chiara Lago, Paweł Pasikowski, Roman Pluta, Anna Sawicka, Przemysław Glaza, David Gisselsson, Samuel Navarro, Rosa Noguera, Joanna Majkut, Paweł Dobrzański, Sylvain Cottens, Michał J. Walczak, Daniel Bexell

**Affiliations:** 1 Translational Cancer Research, Department of Laboratory Medicine, Lund University, Lund, Sweden; 2 Pathology Department, Medical School, University of Valencia-INCLIVA, Valencia, Spain; 3 Centro de Investigación Biomédica en Red de Cáncer, Instituto de Salud Carlos III, Madrid, Spain; 4 Captor Therapeutics Inc, Wrocław, Poland; 5 Division of Clinical genetics, Lund University, Lund, Sweden; 6 Clinical Genetics, Pathology and Molecular Diagnostics, Skåne University Hospital, Lund, Sweden

**Keywords:** GSPT1, MYCN, Neuroblastoma, Targeted protein degradation, Molecular glue, Chemoresistance

## Abstract

**Background:**

High-risk neuroblastoma (HR-NB) is associated with therapy-resistant relapse, and novel therapeutic strategies are needed. GSPT1 is a GTPase involved in protein translation whose disruption may offer therapeutic potential in translation-dependent cancers.

**Methods:**

GSPT1 expression was assessed in publicly available clinical data and tissue microarrays. GSPT1-degrading molecular glues were tested in *MYCN*-amplified NB organoids. Cell viability, cell death assays, western blotting, and proteomics were used to evaluate GSPT1 degraders. Effects on tumor growth and mouse survival were benchmarked against standard-of-care chemotherapy in a chemoresistant NB patient-derived xenograft (PDX) model. RNA sequencing and histopathological analysis were used to assess mechanisms of action in vivo.

**Results:**

*GSPT1* expression is associated with unfavorable outcomes in NB patients. Single-cell analysis revealed elevated *GSPT1* expression in *MYCN*-amplified NB, whereas the E3 ligase *CRBN* (essential for protein degradation) was predominantly expressed in NB cells relative to non-malignant cells. GSPT1-specific degradation decreased cell viability and induced apoptosis in *MYCN*-amplified NB organoids and PDX models. GSPT1 degradation in vivo resulted in NB differentiation and suppression of *MYCN* and its related core regulatory gene networks. In vivo treatment further outperformed standard-of-care chemotherapy and increased survival in a highly chemoresistant NB PDX model.

**Conclusions:**

Inhibition of the translational machinery by GSPT1-degrading molecular glues shows therapeutic potential in chemoresistant *MYCN*-amplified NB.

**Supplementary Information:**

The online version contains supplementary material available at 10.1186/s13046-026-03647-0.

## Background

Neuroblastoma (NB) is a childhood cancer of sympathetic nervous system origin characterized by recurrent genetic alterations including copy number changes (e.g., 1p del, 11q loss of heterozygosity, 17q gain) and mutations (e.g., in *ALK*). *MYCN* is amplified in 20–30% of NBs, and it contributes to several hallmarks of the disease including cell growth, cell survival and inhibition of cell death, differentiation, and metabolism. *MYCN* amplification is a strong predictor of high-risk disease and poor clinical outcomes, and it is often associated with chemotherapy resistance. However, targeting *MYCN* therapeutically has been notoriously difficult [1, 2].

Standard therapy for patients with high-risk NB includes high-dose multimodal chemotherapy, even though it carries a risk of severe and life-long side effects. Many patients initially respond but then relapse with therapy-resistant disease, which carries a poor prognosis. Development of targeted therapies based on molecular alterations is promising and has led to clinical benefits. However, high inter-patient heterogeneity and molecular differences between primary and metastatic/relapsed tumors might hinder successful implementation of targeted therapies in NB [3, 4]. There is therefore an urgent unmet clinical need for the development of therapies that target NB regardless of the mutational landscape, especially for relapsed/chemoresistant disease.

Targeted protein degradation (TPD) is a rapidly developing therapeutic modality that exploits intracellular degradation pathways to destroy disease-driving proteins. TPD works by redirecting E3 ligases to ubiquitinate and degrade target proteins they would not normally recognize or interact with [5, 6], with CRL4^CRBN^ as the best studied ubiquitination system. Several TPD classes with various modes of action have been developed, with proteolysis targeting chimeras (PROTACs) and molecular glues (MGs) advancing in clinical evaluations.

CRBN is expressed in many cell types and in various cellular compartments, making it one of the most versatile ligases for TPD [7]. CRBN-based MGs typically bind to the thalidomide-binding domain of CRBN, in a pocket containing three tryptophan residues. This region allows interactions with the zinc fingers and structurally similar β-hairpin motifs of various proteins. Binding of CRBN to MGs alters the specificity of its interactions, enabling recruitment of entirely different, previously non-interacting proteins or stabilization of weak native protein–protein interactions [8]. This, in turn, leads to target protein ubiquitination and degradation. Although the first MGs were discovered serendipitously, they are also amenable to rational design [9]. MG design is highly dependent on the structure around the binding site, with selectivity arising from differences in amino acid sequences, the protein surface around this binding site, and MG structure. The identification of key stabilizing interactions therefore allows the rational design of MGs, as exemplified by GSPT1 degraders CC-885 and CC-90009 [10, 11].

The small molecular size of MGs helps to overcome limitations of bifunctional degraders (PROTACs), which often exceed Lipinski’s “rules of five” [12], exhibiting low cell permeability and poor oral bioavailability. In contrast, small MG molecules are physicochemically and pharmacokinetically similar to classical inhibitors, with superior drug-like characteristics [13, 14]. Moreover, MGs can degrade proteins lacking a targetable ligand-binding pocket, as their mechanism of action (MoA) depends on direct protein–protein interactions rather than a defined binding site. Therefore, there are several important advantages to using small molecule degraders compared with conventional inhibitors [15]: (i) almost any protein can be degraded, including many targets previously considered undruggable like transcription factors and scaffolding proteins [16]; (ii) due to their event-based MoA, one degrader molecule can degrade multiple targeted protein units, resulting in enzyme-like activity and very high efficacy at low concentrations; and (iii) they might overcome resistance development mediated by mutations in the target protein that threatens clinical efficacy. Supporting their clinical value, MGs such as lenalidomide or thalidomide, which repurpose the CRL4^CRBN^ complex to degrade IKZF1, IKZF3, and CK1α, have now been FDA-approved for the treatment of AML and del(5q) myelodysplastic syndrome (MDS) [17, 18].

*MYCN* amplification is a major event driving NB progression through increased protein synthesis to adapt to increased proliferative needs. The addiction of *MYCN*-amplified NB cells to rapid protein synthesis makes NB highly dependent on protein translation, providing a therapeutic vulnerability. GSPT1 (G1 to S phase transition protein 1), also called eukaryotic release factor 3a (eRF3a), is a crucial component of the protein translation machinery, playing a central role in protein release from ribosomes [19] and nonsense-mediated mRNA decay [20]. Therefore, loss of function or degradation of GSPT1 disrupts protein translation and causes accumulation of misfolded proteins, eventually leading to cellular stress and subsequent cell death.

Here we investigated GSPT1 degradation as a therapeutic strategy in *MYCN*-amplified NB. After describing *GSPT1* and *CRBN* expression in NB patient tumors using bulk and single-cell RNA-seq and protein expression using NB tissue microarrays, we present novel MGs that specifically degrade GSPT1 and induce apoptosis in *MYCN*-amplified NB organoids. In vivo treatment suppressed *MYCN* and associated gene networks and induced substantial NB differentiation. Furthermore, GSPT1 degradation led to strong and persistent anti-tumor effects in chemoresistant patient-derived xenograft (PDX) models of high-risk NB. Overall, our results demonstrate strong preclinical efficacy of GSPT1-targeting MGs, supporting further therapeutic evaluation in high-risk *MYCN*-amplified NB.

## Materials and methods

### Study design

The main goal of this study was to examine GSPT1 as a potential therapeutic target for targeted protein degradation (TPD) with molecular glues (MGs) in chemoresistant HR-NB. Multiple MGs with different target specificity towards GSPT1 were tested in vitro. The initial chemical and biophysical characterization of the compounds was performed by Captor Therapeutics Inc. Then, all compounds were screened in vitro at 10µM to verify their degradation activity. Dose response screen was then performed with the most potent compounds in two different NB organoid models: LU-NB-1 and LU-NB-2. Their effects on GSPT1 degradation as well as cell viability/cell death were compared. At least three biological replicates were performed in each model. Mechanistic analysis was performed in vitro with the two most promising compounds: CTX-56 and CTX-18. In vivo toxicity as well as single treatment was performed with CTX-18. To investigate the mechanisms of action in vivo, short-term studies with compound CTX-18 were carried out using two chemoresistant PDX models: PDX1 and PDX3R. Treated tumors were subjected to histopathological and transcriptional analysis. Long-term survival study was performed using the PDX1 model. The analysis was done with two different compound concentrations and the effects on tumor growth and mice survival were compared with standard-of-care chemotherapy COJEC induction treatment [21]. The end point was set for 150 days after treatment start or tumor size surpassing 1000mm^3^. At least 6 mice were randomly allocated to each group in all studies.

### Cell culture

NB organoids LU-NB-1, LU-NB-2, LU-NB-3R and LU-NB-5 were previously established from high-risk NB PDXs representing primary PDX tumors (PDX1, PDX2, PDX5) and in vivo relapse (PDX3R) [21–24]. NB organoids were grown under serum-free conditions in SCS (Stem Cell) medium, consisting of DMEM medium (Gibco, #21885-025) and GlutaMAX F-12 (Gibco, #31765-027) used 3:1, supplemented with 1% penicillin/streptomycin (Gibco, #15140-122), 2% B27 w/o vitamin A (Gibco, #12587-001), EGF (20 ng/ml, PeproTech, #AF-100-15-500UG) and FGF (40 ng/ml, PeproTech, #AF-100-18B-500UG). Organoids were passaged at least once per week. Before each experiment, organoids were dissociated to single-cell suspension using Accutase (Sigma Alrdich, #A6964) and counted with 0.4% Trypan blue (Invitrogen, #2447851) exclusion using Countess II (ThermoFisher). NB organoids were authenticated using SNP analysis and regularly tested for Mycoplasma. Human Embryonic Kidney 293 (HEK293) cells were cultured in DMEM medium (containing 4.5 g/l glucose, L-glutamine, and sodium pyruvate; Corning, #03222008), supplemented with GlutaMAX™ (Gibco, #35050061), 10% FBS and 1% Penicillin-Streptomycin. Human neuroblastoma Kelly cell line (DSMZ, #ACC 355) was cultured in RPMI 1640 medium (Gibco, #21875-034), 10% FBS (Gibco, #10500-064) and 1% Penicillin-Streptomycin (Biowest, #L0022-100). Hep3B cell line was cultured in EMEM medium (ATCC, #30-2003) supplemented with 10% FBS (Gibco, #10500-064) and 1% Penicillin-Streptomycin (Biowest, #L0022-100).

### RNA extraction, sequencing and analysis

Snap-frozen tumor pieces were incubated at -20 °C in RNAlater-ICE (Invitrogen, #AM7030) for a minimum of 24 h before extraction. RNA extraction was performed using AllPrep DNA/RNA Mini Kit (Qiagen, #80204) according to manufacturer’s instructions. RNA quality was verified using NanoDrop and Qubit. Bulk RNA sequencing was performed by the Center for Translational Genomics, Lund University. Raw counts were normalized with DESeq2 using Human GRCh38 genome sequence (Ensembl database, annotation from GENCODE v33) used as reference. Normalized data was uploaded to and analyzed in R2: Genomic Analysis and Visualization Platform (http://r2.amc.nl). For analysis of patient data, publicly available datasets stored in R2 were used: Tumor Neuroblastoma - Kocak − 649 - custom - ag44kcwolf and Tumor Neuroblastoma - SEQC − 498 - RPM - seqcnb1. For the analysis of transcriptomic PDX data, publicly available datasets [21] stored in R2 were used: Xenograft Neuroblastoma COJEC invivoPDX123 (20210601) - Aaltonen − 120 - custom - gencode33- for transcriptomic analysis of different PDX tissues; Tumor Neuroblastoma COJEC_PDX3 Organoids - Manas − 24 - custom - gencode33- for the transcriptomic analysis of PDX-derived organoids representing cured and relapsed tumors. One way analysis of variance (ANOVA) with Welch correction was used to calculate the *p* values.

### GSEA analysis

Raw sequencing data was processed as described in RNA-seq Data processing section in Supplementary Materials and Methods.

Gene set enrichment analysis (GSEA) was conducted using a pre-ranked list derived from the differential expression results, ranked by the metric sign(log₂FoldChange) × –log₁₀(*p*-value). Enrichment was performed with clusterProfiler (v4.12.6) [25], using gene sets obtained from the Molecular Signatures Database (MSigDB) via the msigdbr package (v7.5.1) and mapped to gene identifiers using org.Hs.eg.db (v3.19.1). The default parameters of the GSEA function were slightly adjusted to improve specificity, using maxGSSize = 2000, *p*valueCutoff = 0.05, eps = 0, and nPermSimple = 10,000. Enriched gene sets were considered meaningful if they met a false discovery rate (FDR) threshold of less than 5%.

### Spatial transcriptomics and scRNA analyses in patient datasets

Expression of *GSPT1*, *CRBN*, and *NTRK1* was analyzed using a published Visium spatial transcriptomics (ST) dataset from high-risk NB adrenal tumors [26]. Gene expression visualization was based on the ALRA-imputed assay matrix to reduce dropout effects and enhance spatial signal detection.

Furthermore, expression of *GSPT1* and *CRBN* was analyzed in two independent public NB scRNA datasets, from Bedoya-Reina et al. [27] and Bonine et al. (NBAtlas [28]), consisting of 11 and 68 tumors respectively.

All analysis were based on original Seurat objects from the respective studies, with no additional data processing.

### Cell viability and cell death assays

LU-NB-1, LU-NB-2 and LU-NB-3R organoids as well as HEK293 cells were seeded as single cell suspension in opaque 96-well plates (Corning, #3610), at a density of 5000 cells per well in a total volume of 90µl/well. Cells were incubated for 24 h to allow organoid formation prior to the treatment. Organoids were treated with tested MGs at a range of concentrations: 0.1 nM-10 µM in a total volume of 100µl/well. Technical triplicates were used for each concentration. Organoids were incubated with the compounds for 72 h or 7 days (re-treatment was performed after 72 h). For combination treatment with pan-caspase inhibitor, Z-VAD (OMe)-FMK (Santa Cruz Biotechnology, #sc-311561 A), 50 µM working concentration was added four hours before treatment with MGs for the next 48 h. Cell viability and cell death were measured using CytoToxGlo kit (Promega, #G9292) according to the manufacturer’s instructions. Luminescence was measured with a Synergy2 Multi-Mode plate reader (BioTek). Raw luminescence signals from the DMSO wells were averaged (DMSO = 100%) and each compound treated well was normalized to DMSO (calculated as % of DMSO). At least three biological replicates were used for statistical analysis.

### Caspase 3 assay

Single cells were seeded into 6-well plates at a density of 0.5 × 10^6^ cells per well and were left to grow and reassemble for 24 h. Cells were then treated with MGs at 1 µM concentration, using DMSO as a negative control. To assess apoptosis in real time, 24, 48 and 72 h post-treatment, 0.2% NucView 530 Caspase-3 dye (Biotium, #10406) was added to the cells (3 µl per well). Imaging was performed using an inverted microscope (Zeiss AX10, serial number 3847001274).

### Transfection

Cells were seeded in 6-well plates at a density of 0.5 × 10^6^ cells per well, in 1.5 mL serum- and antibiotic-free SCS medium. The following day, cells were transfected with siRNA targeting CRBN (SMARTPool, Dharmacon™, #L-021086-00-0005)*)* or non-targeting siRNA (Dharmacon™, #D-001810-10-05) using Lipofectamine RNAiMAX *(*Invitrogen, #B778-100*)* according to manufactures standardized protocol. 7.5 µL of Lipofectamine was used per each condition. Working concentration of 25 nM of siCRBN or siNegative was used. OptiMEM (Gibco, #31985-062) was used for siRNA and Lipofectamine dilution. The siRNA-lipid complexes were added dropwise to each well and were incubated with the cells for 48 h.

### AnnexinV/PI staining

LU-NB-1 and LU-NB-2 organoids were dissociated to single cells using Accutase as described in cell culture section and seeded at the density of 0.5 × 10^6^ cells/well in a 6-well plate. 24 h later, organoids were treated with either 100 nM or 1 µM MG or a control amount of DMSO and were allowed to grow for further 48 h. Organoids were then dissociated, resuspended in AnnexinV binding buffer (Invitrogen, #00-0055-43) and stained with Annexin V/propidium iodide (AnnexinV: Invitrogen, #11-8005-72, PI; Invitrogen, #00-6990-50) for 15 min. Flow cytometry was performed using FACS Melody™ Cell Sorter (BD Biosciences), and data were analyzed using FlowJo 10.8.1 software. At least three biological replicates were performed.

### Western blotting

LU-NB-1, LU-NB-2 and LU-NB-3R organoids were dissociated using Accutase as described in cell culture section and 0.5 × 10^6^ single cells resuspended in 2 ml of SCS medium were seeded per each well of a 6-well plate. The next day cells were treated with MGs at a range of concentrations 0.1 nM-10 µM for the following 24 h. For MG132 co-treatment, organoids were treated with MG132 *(*Sigma Aldrich, #M7449*)* at a concentration of 5 µM for 1 h before treatment with MG (10 nM, 100 nM) for the following 6 h. After that, cells were collected from each well and the pellets were lysed in RIPA buffer (ThermoFisher, #89900) supplemented with protease inhibitor (1:25, Roche, #04693116001). Protein concentration was evaluated using Pierce™ BCA protein assay kit (Thermo Scientific™, #323225). Samples were separated on 4–20% gradient SDS-polyacrylamide mini-PROTEAN TGX precast gels (4–20%, Bio-Rad, United States, #4561096) and transferred onto nitrocellulose membranes using semi-dry turbo system (BioRad, #1704271). Membranes were then blocked with 5% milk/PBS-T for 1 h at RT and incubated with primary antibodies O/N at 4 °C. All antibodies were diluted in blocking buffer (5% milk in PBS-T). Signal was developed using Luminata Forte Western HRP substrate (MilliporeSigma, #WBLUF0500) and imaging was done with Amersham Imager 600 (GE Healthcare Bio-Sciences AB). Used antibodies: GSPT1 (1:1000; Invitrogen, #PA5-28256), CRBN (1:1000; Invitrogen, #PA5-61122), MYCN (1:1000; Cell Signaling, #D4B24), PARP (1:1000; Cell Signaling, #9542), SALL4 (1:2000; Abcam, #ab57577), IKZF1 (1:2000, Proteintech, #12016-1-AP), CSKNA1 (1: 2000; Abcam, #ab206652). Actin (HRP-conjugated, 1:10 000, Invitrogen, #MA5-15739-HRP) and GAPDH (1:5000; RD, #2275-PC-100) were used as loading controls. HRP-conjugated secondary antibodies; anti-mouse (1:10 000, 2 mg/ml, Abcam, #GR3395730-8) and anti-rabbit (1:10 000, 2 mg/ml, Abcam, #GR3399203-17) were applied for 1 h at RT. At least three biological replicates were performed for each experiment. Data was analysed using ImageJ and ImageLab softwares. Statistical analysis of the results is shown in Supplementary Data Files S2 and S3.

### Immunohistochemistry and histological analyses

Fixed tumor samples were embedded in paraffin and sliced into 4 μm sections using Leica SM2000R microtome. Tissues were baked at 62 °C for 2 h. Histopathological analysis was performed using H&E staining. Deparaffinization of the tissues (serial incubations in xylene and EtOH solutions (100%, 95% and 70%)) was followed by 3 min haematoxylin staining and 2 min eosin counterstain. Stained slides were dehydrated and mounted using xylene-based mounting medium (Sigma Aldrich, #03989).

Immunohistochemical DAB (3,3’-Diaminobenzidine) staining was performed manually. Following tissue deparaffinization, heat-induced antigen retrieval was performed using sodium citrate buffer (pH 6.0). 0.3% H_2_O_2_ was used to block internal peroxidase activity. Tissues were then blocked for 1 h at RT using 4% BSA/PBS-T (0.1% Tween) and incubated with primary antibodies overnight at 4 °C. All antibodies were diluted in 2% BSA/PBS-T (0.05% Tween). The antibodies used: GSPT1 (1:500; Invitrogen, #PA5-28256), CRBN (1:500; Invitrogen, #PA5-61122), PHOX2B (1:1000; Abcam, #ab183741), KI67 (1:200; Abcam, #ab16667), NTRK (1:500, Invitrogen, #MA5-32123). HRP-conjugated secondary antibody solutions (Cell Signaling; anti-rabbit: #8114S, anti-mouse: #8125S) were used for 1 h at RT for. ImmPACT DAB solution (Vector, #SK-4105) was applied for 5 min and nuclear counterstain was done using haematoxylin stain. Slides were mounted using xylene-based mounting medium (Sigma Aldrich, #03989). Images were taken using NanoZoomer slide scanner (Hamamatsu). Processing and quantification of images was done using QuPath 0.2.3 and NDPView2. Quantification of stainings was performed using five different tumors per condition. Histopathological analysis of TMAs was performed blindly by clinical pathologists. The evaluation of GSPT1 and CRBN expression intensity in PDX tumors was performed blindly based on 17 (GSPT1) and 13 (CRBN) tumors representing 3 PDX models.

### TUNEL staining

Tissues were stained for the presence of apoptotic cells using TUNEL assay kit (Abcam, #ab206386) according to the manufacturer’s instructions. In short, following the deparaffinization, tissues were permeabilized using Proteinase K (1:100). The activity of endogenous peroxidases was then blocked using 3% H_2_O_2_. Tissues were then equilibrated and labelled using TdT enzyme. After 1.5 h, the reaction was stopped and the tissues were blocked using blocking buffer and prepared for detection by incubation in diluted conjugation buffer. The apoptotic cells were detected using DAB staining. Methyl Green was used as a counterstain. Stained tissues were dehydrated and mounted using xylene-based mounting media (Sigma Aldrich, #03989). For quantification, four different tissues were analysed per condition. Images were taken using NanoZoomer slide scanner (Hamamatsu).

### Proteomic sample preparation

NB PDX organoids were treated with the MG at 10 nM or 1 µM concentration. The cells were collected after either 6 or 24 h of treatment. DMSO was used as a control. Obtained cell pellets were further prepared using S-trap micro columns according to manufacturer’s protocol with minor changes. 100 µL of lysis buffer (8% SDS in 50 mM TEAB with addition of protease and phosphatase inhibitors, pH = 8.5) was added to each pellet. Complete cell lysis was obtained by ultrasonication using SONOPULS GMmini20 sonicator (Bandelin) equipped with MS 1.5 probe over 2 min, with 30% power pulses lasting 1 s with 4 s pauses. Protein concentrations in lysates were determined using Pierce™ Quantitative Colorimetric Protein Assay (Thermo Scientific) and volumes corresponding to 50 µg of protein (usually around 20 µL) were transferred to new LoBind tubes (Eppendorf). Appropriate volume of lysis buffer was added to every sample to reach 26 µL total volume. Next, samples were reduced by addition of 1.1 µL of 120 mM TCEP in water and incubation in 55 °C for 15 min, alkylated by addition of 1.3 µL of freshly prepared 500 mM iodoacetamide in 50 mM TEAB and incubation for 20 min in RT in darkness, and acidified by addition of 2.8 µL of 27.5% phosphoric acid. Then 185 µL of binding buffer (100 mM TEAB in 90% methanol, pH = 7.55) was added, samples were transferred to the S-trap micro columns placed in 2 mL container tubes and centrifugated in 4000 x g for 1 min. Subsequently, S-trap resins were washed 4 times by addition of 150 µL of binding buffer and centrifugation in 4000 x g for 30 s. Flow-through was discarded and resins were dried by additional centrifugation (4000 x g for 1 min). Flow-through containers were replaced with new LoBind tubes and 20 µL of digestion solution (0.125 µg/µL trypsin/Lys-C mixture (Thermo Scientific) in 50 mM TEAB) was carefully added on top of the resin, making sure that no air bubbles were trapped on top of the resin. Columns were sealed and incubated in 37 °C overnight. The next day peptides were eluted from the resin by subsequent washing with 40 µL of three eluting buffers (50 mM TEAB in water, 0.2% formic acid in water, and water: acetonitrile 1:1 (v/v)) and centrifugation (4000 x g for 1 min). Resulting peptide mixtures were freeze-dried and reconstituted in 100 µL of 0.1% formic acid in water. Peptide concentration was determined using Pierce™ Quantitative Colorimetric Peptide Assay (Thermo Scientific) and 30 µL of each sample was transferred to high recovery plastic HPLC vial with built-in inserts (Phenomenex). Volume of the sample corresponding to 1 µg of peptide mixture was then used for LC-MS injection.

### LC-MS DIA measurements

Data acquisition was performed on Exploris 480 (Thermo Scientific) mass spectrometer coupled with Dionex UltiMate 3000 RSLCnano chromatograph. Chromatographic separation was carried out on an Acclaim PepMap 100 C18 analytical column (250 mm length, 0.075 mm ID and 3 μm particle size) (Thermo Scientific) using 4-minute peptide trapping on a PepMap Neo Trap Cartridge (5 mm x 30 μm) (Thermo Scientific) in reversed 40 µL/min flow of loading solution (0.1% formic acid in water). Mobile phases A (0.1% formic acid in water) and B (acetonitrile: water 80:20 (v/v) with 0.1% formic acid) were introduced with flowrate of 300 nL/min in following gradient: 0 min – 5% B, 5 min – 5% B, 95 min – 36% B (main separation), 97 min – 95% B, 113 min – 95% B (column washing), 115 min – 5% B, 130 min – 5% B (column equilibration). Column oven was set to 30 °C. Samples were introduced to Nanospray Flex ion source via stainless steel emitter (Thermo Scientific) to Nanospray Flex ion source using capillary voltage of 2 kV and capillary temperature of 275 °C. The spectrometer was calibrated externally (SD < 3 ppm) using Pierce FlexMix calibration solution (Thermo Sceintific) and operated in positive ion mode according to DIA strategy. Single MS full scan (120 000 Orbitrap resolution, scan range 350–1400 m/z, RF lens 70%, AGC target 300%, and max. injection time 45 ms) was followed by 48 DIA scans (precursor mass range 361–1033, isolation window of 14 Th with 1 Th overlap, normalized CE 28%, orbitrap resolution of 15 000, RF lens 70%, AGC target 1000%, and max. injection time of 25 ms). All data were collected at speed of 40 Hz as peak centroids, starting at 10 min and ending in 105 min of chromatographic separation.

### LC-MS data analysis

Resulting RAW files were further processed using DIA-NN software package (version 1.9). Library free search, utilizing deep learning-based spectra and RT prediction, was performed using canonical *Homo sapiens* (taxID = 9606) sequences downloaded from UniProtKB on Aug 14th, 2024. Other key parameters of search were set as follows: protease – Trypsin/P with missed cleavages set to 1; max number of variable modifications – 3; fixed modifications: C carbamidomethylation; variable modifications: N-term M excision, oxidation (M) and acetylation (N-term); peptide length range: 7–30 residues; precursor charge: 2–6; precursor m/z range: 350–1500; fragment ion m/z range: 200–2000. Mass accuracy was set to 10 ppm in MS mode and 20 ppm in MS/MS mode. MBR (Match Between Runs) and “no shared spectra” options were enabled, whereas “heuristic protein inference” was disabled. Search was performed in single-pass mode with QuantUMS (high precision) quantification strategy and RT-dependent cross-run normalization.

DIA-NN search results with missing values in any of the samples were filtered out. Resulting protein matrix was log2-transformed and *P*-values (for each sample vs. control) were calculated using background based t-test. The results comparing each sample with the controls were presented on volcano plots.

### Animal experiments

NB PDX models were created using NB PDX-derived organoids, described in cell culture section of Materials and Methods. NB PDX organoids were dissociated using Accutase and 1 × 10^6^ cells were resuspended in a 100 ml mixture of stem cell medium and Matrigel (Corning, #354234) used 3:1. Prepared cell solution was injected subcutaneously into the flanks of in-house bred female NSG mice. After the tumors reached appropriate tumor size, mice were randomly allocated to the corresponding treatment groups. 6 mice were allocated to each group for statistical analysis. Tumor size was measured using a digital caliper and calculated according to the formula V = (length x width^2^) x 3,14/6 mm^3^. All mice were monitored 3 times/week for weight loss and general signs of well-being. Mice were euthanized based on tumor size, weight loss, overall health or end of study time. CTX-18 was resuspended in vehicle (55% saline, 40% PEG40, 5% Kolliphor HS) and was administered by oral gavage. COJEC drugs (Cisplatin: Santa Cruz Biotechnology, #sc-200896, Vincristine: Santa Cruz Biotechnology, #sc-201434, Cyclophosphamide: Santa Cruz Biotechnology, #sc-361165, Etoposide: Santa Cruz Biotechnology, sc-357357, Carboplatin: Santa Cruz Biotechnology, sc-202093) were dissolved in saline and were administered via intraperitoneal injections. Control mice received vehicle by oral gavage. At the experimental endpoint, all animals were examined for potential metastases and signs of toxicity. All in vivo procedures were conducted according to the guidelines from the Swedish Ethics Committee for Animal Research (permit nr 19012-19 and 15579-24). The maximal tumor size permitted by the ethics committee was 2000 mm³, and this limit was not exceeded in any of the experimental groups.

### Survival study

NSG mice were randomly allocated to treatment groups (CTX-18 30 mg/kg, CTX-18 10 mg/kg, COJEC chemotherapy or control) when the tumors reached the size > 200mm^3^. All mice were treated for 3 weeks or until mice reached euthanization criteria. Mice were treated with the MG 5 days/week by oral gavage. Mice in the COJEC group were treated 3 times/week by i.p. injections (Monday: 1 mg/kg cisplatin, 0.25 mg/kg vincristine, Wednesday: 75 mg/kg cyclophosphamide, 4 mg/kg etoposide, Friday: 25 mg/kg carboplatin). Tumors were measured and mice weighted 3 times per week. Mice were sacrificed when tumors reached 1000mm^3^ or after 150 days from the treatment initiation. Tumors were resected and tumor pieces were snap-frozen or fixed in 4% paraformaldehyde. If no tumor was available for resection, livers, lungs and femurs were collected for evaluation of the presence of potential metastases.

### Short-term study

NSG mice bearing PDX1 tumors were randomly allocated to treatment groups (CTX-18 30 mg/kg or control) when the tumors reached the size ~ 400-500mm^3^. Compounds were administered by oral gavage either as a single dose at day 1 or for 5 days (3 doses given at day 1,3 and 4) with CTX-18, control mice were dosed with vehicle for 5 days (3 doses). Mice bearing PDX3R tumors were randomly allocated to treatment groups (10 mg/kg CTX-18 and 30 mg/kg CTX-18) or control when tumors reached the size ~ 300mm^3^. Mice were dosed by oral gavage 4 times over 7 days (given at day 1,2,4 and 6) and the study was terminated at day 7. Tumor volumes were measured before each dosing. After the appropriate time, mice were sacrificed and resected tumors were divided and snap-frozen or fixed in 4% formaldehyde for further analysis.

### Statistical analysis

Data was analyzed using GraphPad Prism v10.2.3. Non-linear regression analysis was used for all the dose-response data to the IC_50_ and DC_50_ values. Two-way ANOVA was used to determine significance. In vivo groups were compared using one-way ANOVA followed by Tukey’s multiple comparison test on the last day of all mice being alive. Survival analysis was done using Kaplan-Meier analysis and log-rank tests were applied between indicated groups. Data from RNA-seq (R2 software) was analyzed using one-way ANOVA with a 0.01 FDR correction for multiple testing or Welsh’s one-way ANOVA. The *p*-value of < 0.05 was considered significant.

## Results

### High GSPT1 expression is associated with poor prognosis in *MYCN*-amplified NB

**Fig. 1:**
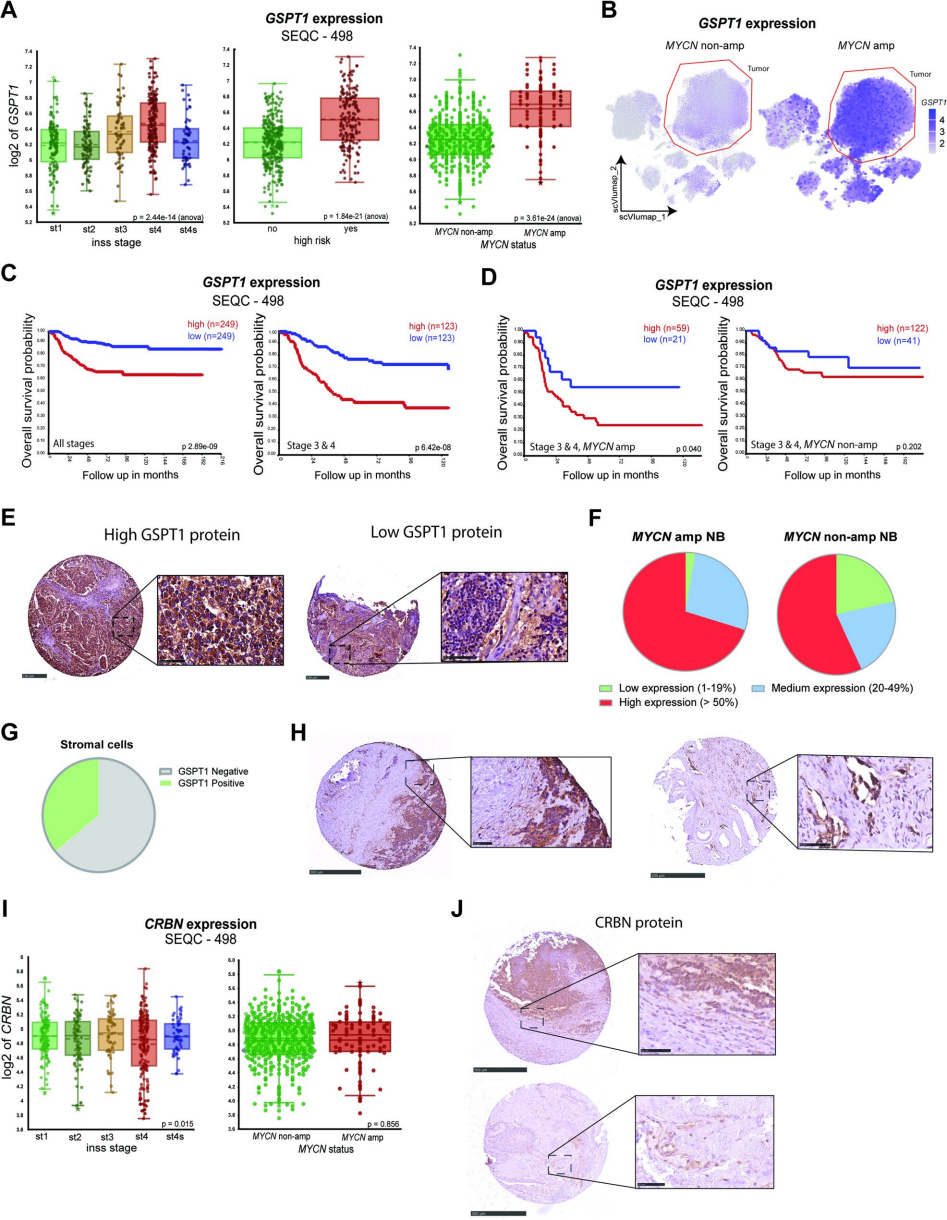
GSPT1 expression is associated with poor prognosis in high-risk NB **A**) *GSPT1* mRNA expression in the SEQC (n=498) NB patient dataset. From left to right: box plots comparing *GSPT1* expression based on INSS stage, risk status, and *MYCN* amplification. Log2 expression of *GSPT1* is shown on the y-axis. One-way ANOVA was used for statistical analysis. Asterisks indicate outliers; **B**) Single-cell analysis of *GSPT1* expression in clinical NB patient tumors with and without *MYCN*-amplification, data from NBAtlas (n=68, [28]), tumor cells are outlined in red; **C**) Survival of patients with NB expressing high and low gene expression of *GSPT1* in the SEQC498 dataset, presented using Kaplan-Meier survival curves. Full patient set (left) and stage 3 and 4 patients (right) (red, high *GSPT1* expression, blue, low *GSPT1*, log-rank test, median used as cut-point for analysis); **D**) Survival of NB patients stratified by *GSPT1* gene expression levels and *MYCN* amplification status. Data obtained from the SEQC498 dataset and presented using Kaplan-Meier survival curves (red, high *GSPT1* expression, blue, low *GSPT1*, log rank test, first quartile used as cut-point for analysis); **E**) Representative photomicrographs of IHC staining of NBs with high (left) and low (right) GSPT1 expression (scale bar: 250 and 50 µm); **F**) Pie charts showing distribution of samples on the TMA exhibiting high (>50%), medium (20-49%), and low (<20%) GSPT1 expression in *MYCN*-amplified (n=40, left) and* MYCN* non-amplified (n=28, right) tumors; **G**) Pie chart showing the distribution of samples on the TMA with and without GSPT1 expression in stromal cells (n=68); **H**) Left: representative photomicrographs of IHC staining of NBs showing GSPT1 expression in tumor tissue (scale bars: 500 and 50 µm); right: representative photomicrographs of IHC staining of NBs showing GSPT1 expression in endothelial cells (scale bar: 500 and 50 µm); **I**) *CRBN* mRNA expression in NB patient tumors within the SEQC (n=498) dataset. From left to right: box plots comparing mRNA expression based on INSS stage and *MYCN* amplification status. Log2 expression of *CRBN* is shown on the y-axis. One-way ANOVA was used for statistical analysis. Asterisks indicate outliers; **J**) Upper panel: representative photomicrographs of IHC staining of CRBN in NBs compared to stromal compartments (scale bars: 500 and 50 µm); Lower panel: representative photomicrographs of IHC staining of CRBN in endothelial cells (scale bars: 500 and 50 µm)

We first explored gene expression of *GSPT1* in NB patient tumors by analyzing public databases (SEQC (*n* = 498), Kocak (*n* = 649); R2 platform). High mRNA expression of *GSPT1* was strongly associated with advanced stage, high-risk tumors, and *MYCN* amplification (Fig. 1A, Supplementary Fig. 1A). Single-cell mRNA analysis of patient tumors (NBAtlas [28]), additionally confirmed increased *GSPT1* expression in tumor cells with *MYCN* amplification (Fig. 1B). Further analysis revealed that *GSPT1* expression was associated with worse overall survival in patients with NBs, especially in patients with stage 3–4 NB (Fig. 1C, Supplementary Fig. 1B). The association was particularly strong in a subset of patients with the highest *GSPT1* expression (Supplementary Fig. 1C). Moreover, the effect was more pronounced in stage 3–4 patients with *MYCN* amplification compared with patients with non-*MYCN*-amplified tumors (*p* = 0.040 for *MYCN* amplified vs. *p* = 0.202 for *MYCN* non-amplified; Fig. 1D), suggesting the importance of GSPT1 in *MYCN-*amplified high-risk (HR)-NB.

To evaluate GSPT1 protein levels in NB patient tumors, we performed immunohistochemistry and analysis using NB tissue microarrays (TMAs) covering 68 NBs (5 non-HR, 63 HR; 28 *MYCN-*non-amplified, 40 *MYCN*-amplified).

GSPT1 protein was detected in all NB tumors, with the majority (65%) showing high expression levels (present in > 50% of tumor cells). Fewer than 10% of tumors displayed low GSPT1 expression (1–19% of tumor cells) (Fig. 1E).

Notably, among *MYCN*-amplified tumors, only one case (< 3%) exhibited low GSPT1 expression, whereas the remaining tumors showed medium (27%) or high (70%) expression levels, suggesting a potential association between GSPT1 expression and *MYCN* amplification status. Consistent with this, six of seven tumors with low GSPT1 expression were *MYCN* non-amplified (Fig. 1F). While GSPT1 expression was predominantly localized to tumor cells, additional staining was observed in spindle-shaped stromal and/or endothelial cells in 36% of cases (Fig. 1G and H).

Gene ontology analysis of the 1000 most differentially expressed genes between tumors from HR and low-risk NB patients (R2 platform, SEQC dataset, *n* = 498; Supplementary Material 1) revealed that HR-NB was most enriched for pathways associated with protein translation (Supplementary Fig. 1D), as were *MYCN*-amplified NB compared with *MYCN*-non-amplified tumors (Supplementary Fig. 1E). These data suggest that protein translation might be a therapeutic vulnerability in *MYCN*-amplified HR-NB.

Thus, GSPT1 is highly expressed at gene and protein levels in patient NB tumors and its high expression is associated with aggressive, high-risk *MYCN*-amplified NB and poor prognosis.

### CRBN expression in NB patient tumors

A prerequisite for MG-mediated degradation is expression of the coopted E3 ligase, in this case Cereblon (CRBN). Analysis of public datasets using the R2 platform showed abundant and uniform expression of *CRBN* in different NB patient cohorts, regardless of *MYCN* status or disease stage (SEQC *n* = 498, Fig. 1I). Analysis of NB TMAs revealed that CRBN protein was expressed in 96% of analyzed NBs (63% of NBs high expression, 24% medium expression, and 9% low expression). In addition, CRBN expression appeared to be relatively tumor cell-specific, with lower expression observed in non-malignant tumor compartments, e.g. endothelial cells (24% of NB cases), spindle-shaped stromal cells (26% of NB cases) (Fig. 1J).

### Single-cell gene expression of GSPT1 and CRBN in NB patients´ tumors

**Fig. 2:**
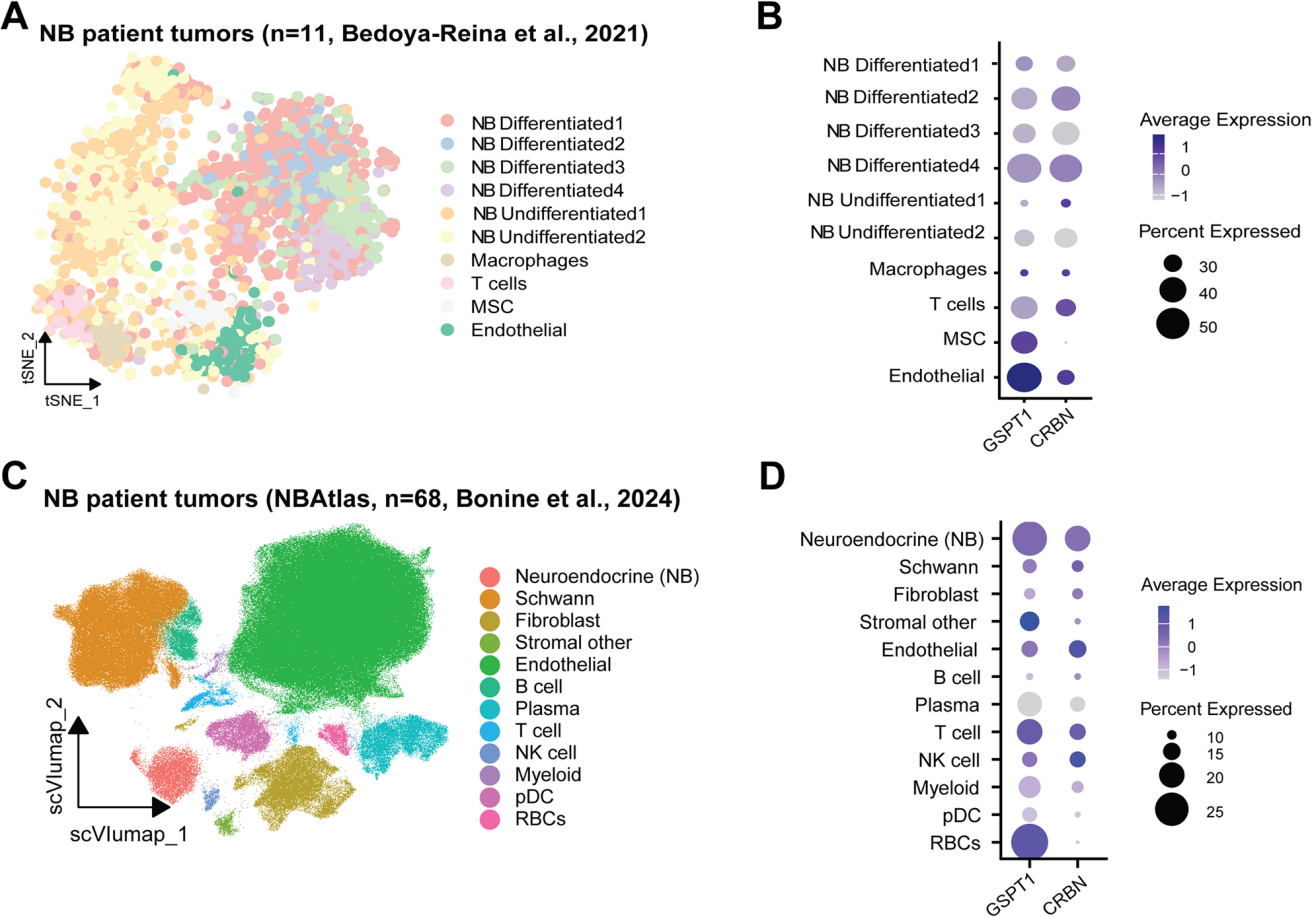
Single-cell expression of *GSPT1* and *CRBN* in neuroblastoma patient tumors. **A** Distribution of NB tumor cells (NB Differentiated 1–4, NB Undifferentiated 1–2) and non-malignant cells (T cells, macrophages, endothelial cells, and MSCs) in NB patient tumors (*n* = 11, Bedoya-Reina, [27]), visualized by tSNE plot; **B**) *GSPT1* and *CRBN* gene expression in NB cells and non-malignant cells in NB patient tumors. Data from Bedoya-Reina; **C**) Distribution of NB tumor cells (Neuroendocrine) and non-malignant cells in NB patient tumors (NBAtlas, *n* = 68, [28]), visualized by UMAP plot. DC - dendritic cells, RBCs - red blood cells; **D**) *GSPT1* and *CRBN* gene expression in NB cells and non-malignant cells. Data from NBAtlas

We next explored *GSPT1* and *CRBN* expression in NBs using scRNA-seq data (Bedoya-Reina et al. [27]). *GSPT1* mRNA was expressed in the majority of NB cell clusters, with the highest expression in tumor cells, mesenchymal stem cells, and endothelial cells (Fig. 2A, B) and very low *GSPT1* expression in macrophages. There was high expression of *CRBN* in NB tumor cells and relatively low *CRBN* expression in surrounding non-tumor cells (Fig. 2B). Analysis of data from the NBAtlas [28], containing a broader repertoire of patient tumors, confirmed *GSPT1* expression in both tumor and stroma cells and relatively low *CRBN* expression in non-malignant cells (Fig. 2C, D). These findings suggest that CRBN-mediated GSPT1 degradation could preferentially target *MYCN*-amplified NB cells because of the relatively low *CRBN* gene expression in the non-malignant compartment.

### GSPT1 and CRBN are expressed in NB PDX models

**Fig. 3:**
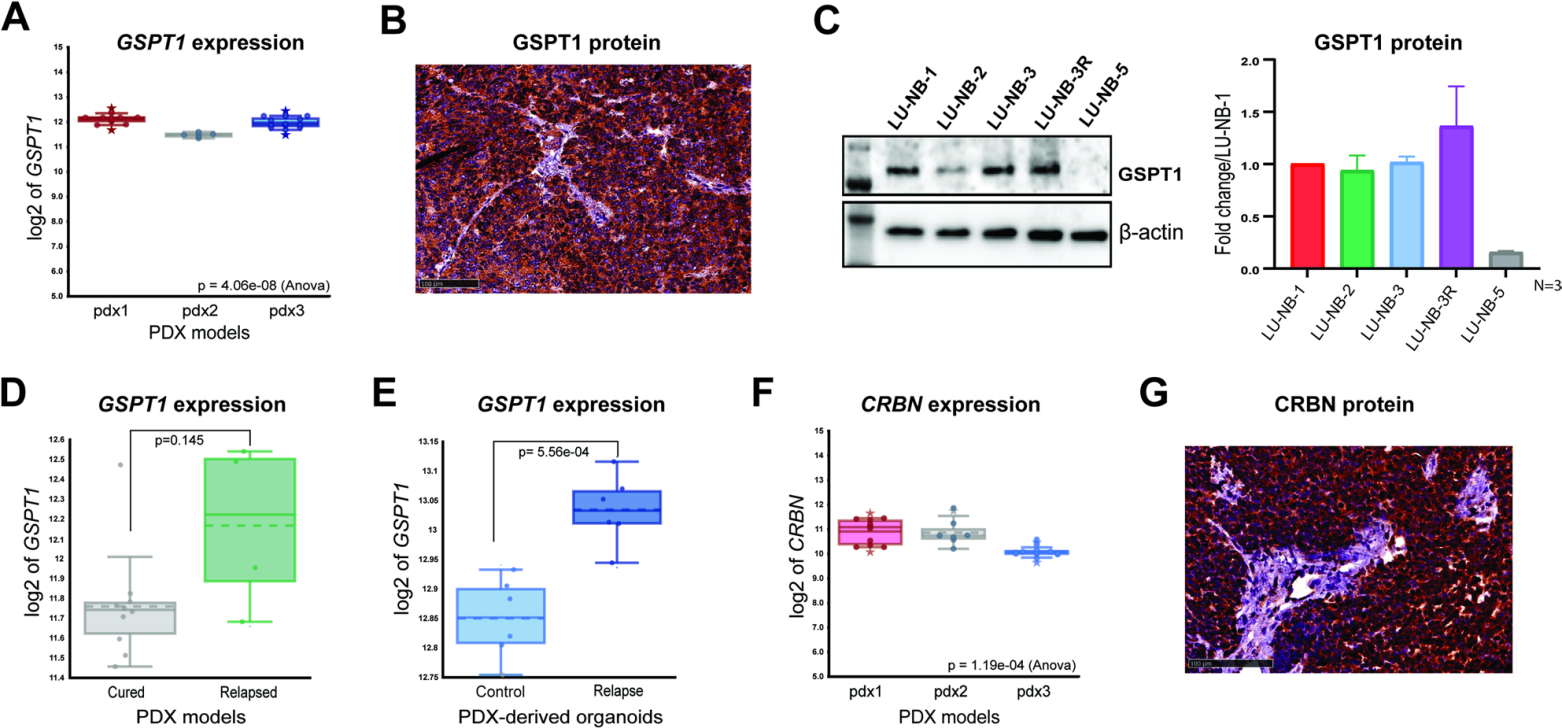
GSPT1 and CRBN expression in patient-derived neuroblastoma models. **A ***GSPT1* mRNA expression in *MYCN*-amplified NB PDX models. One-way ANOVA was used for statistical analysis. Asterisks indicate outliers; **B**) Representative photomicrograph of IHC showing GSPT1 protein expression (high intensity) in a *MYCN*-amplified PDX model (scale bar: 100 μm); **C**) Western blot analysis of GSPT1 protein expression in *MYCN*-amplified NB PDX-derived organoids LU-NB-1, LU-NB-2, LU-NB-3, and LU-NB-3R and the *MYCN*-non-amplified LU-NB-5 model. Quantification was based on triplicates; **D**) Comparison of *GSPT1* mRNA expression between PDX3 tumors that fully responded to COJEC chemotherapy (cured) and tumors that acquired resistance and regrew (relapse). Analysis was performed in R2 using publicly available data [21]. *t*-test with Welch correction was used for statistical analysis; **E**) Comparison of *GSPT1* mRNA expression between NB organoids derived from untreated PDX3-tumors (Control) and PDX3-tumors that relapsed after chemotherapy (Relapse). Analysis was performed in R2 using publicly available data [21]. *t*-test with Welch correction was used for statistical analysis; **F**) *CRBN* mRNA expression in *MYCN*-amplified NB PDX models. One-way ANOVA was used for statistical analysis. Asterisks indicate outliers; **G**) Representative photomicrograph of IHC image showing CRBN protein expression (high intensity) in a *MYCN*-amplified PDX (scale bar: 100 μm)

We next investigated GSPT1 and CRBN expression in *MYCN*-amplified NB PDX models and PDX-derived 3D tumor organoids (Supplementary Fig. 1F, G; [21–24]). Using the R2 platform to analyze publicly available datasets [21], we identified abundant *GSPT1* gene expression in all *MYCN*-amplified NB PDX models (Fig. 3A). Scoring of GSPT1 IHC in NB PDX tumors (*n* = 17 across 3 PDX models) confirmed abundant GSPT1 protein expression, with 70% of tumors exhibiting high and 30% medium staining intensity (Fig. 3B), without apparent variation in intensity between different PDX models. Western blot analysis confirmed GSPT1 protein expression in *MYCN*-amplified organoids (Fig. 3C) and minimal/very low GSPT1 expression in organoids derived from a *MYCN*-non-amplified PDX model (LU-NB-5). Additionally, there was a trend towards increased *GSPT1* expression in PDXs derived from tumors that had relapsed after COJEC chemotherapy [21] (Fig. 3D) and in organoids derived from relapsed PDX tumors at both mRNA and protein levels (Fig. 3C, E [21]). Similarly, mRNA analysis of *MYCN*-amplified PDXs and PDX-derived organoids revealed *CRBN* gene expression across all models (Fig. 3F), including relapsed organoids (Supplementary Fig. 1G). CRBN protein expression was also demonstrated in PDX tumor tissues (Fig. 3G), with 83% of analyzed tumors showing high and 17% medium staining intensity (*n* = 13 across 3 PDX models).

Collectively, analysis of NB PDXs and PDX-derived organoids revealed robust expression of GSPT1 and CRBN in models representing both primary and relapsed *MYCN*-amplified tumors.

### Characteristics of GSPT1-degrading molecular glues

Optimization of MG degraders through an iterative structure-activity relationship approach led to the discovery of several compounds specifically targeting GSPT1. We evaluated nine of such MG degraders. Three of the MGs demonstrated both potent GSPT1 protein degradation (WB analysis) and efficacy (viability assay) in NB-derived organoids (Supplementary Fig. 2A). We selected two MGs: CTX-56 and CTX-18 (Supplementary Fig. 2B), for their superior efficacy (with the third, CC-90009, being the reference compound, the first GSPT1 degrader to enter clinical evaluation). These quinoline-based CRBN binders exhibited excellent selectivity, with no activity against typical CRBN neotargets like Sall4. Their design and optimization were aided by molecular modeling of CRBN-MG-GSPT1 ternary complexes, which provided insights into the crucial binding interactions and conformational preferences driving the observed activity and specificity.

Biophysical analysis confirmed ternary complex formation between GSPT1, CRBN, and each of the compounds, showing good kinetic parameters with similar dissociation constants (Kd) and stronger binding compared to the reference compound CC-90009 (Supplementary Fig. 2C). Moreover, in-cell ternary complex formation was superior to CC-90009 for CTX-18 in cells expressing WT GSPT1, while no binding was observed when mutant GSPT1 was expressed (Supplementary Fig. 2D). No signal was observed in the presence of lenalidomide, a CRBN-recruiting MG targeting IKZF1. These data not only confirm ternary complex formation occurring in cells but also suggest specificity of CTX-18 towards GSPT1. Accordingly, strong in-cell GSPT1 degradation was demonstrated in HEK293 with HiBiT-tagged GSPT1 following a 6 h treatment with the tested compounds, an effect that could not be attributed to cell death (Supplementary Fig. 2E). Both compounds showed efficacy comparable to the reference compound CC-90009, with similar DC_50_ values (the concentration needed to degrade 50% of the target protein) and maximal degradation (Supplementary Fig. 2F). Importantly, both MGs effectively degraded GSPT1 and reduced viability of NB Kelly cells (Supplementary Fig. 2G, H), exhibiting inhibitory concentration (IC_50_) values of 29 nM (CTX-56) and 14 nM (CTX-18).

### Specific GSPT1 degradation is highly effective in HR-NB organoids

**Fig. 4:**
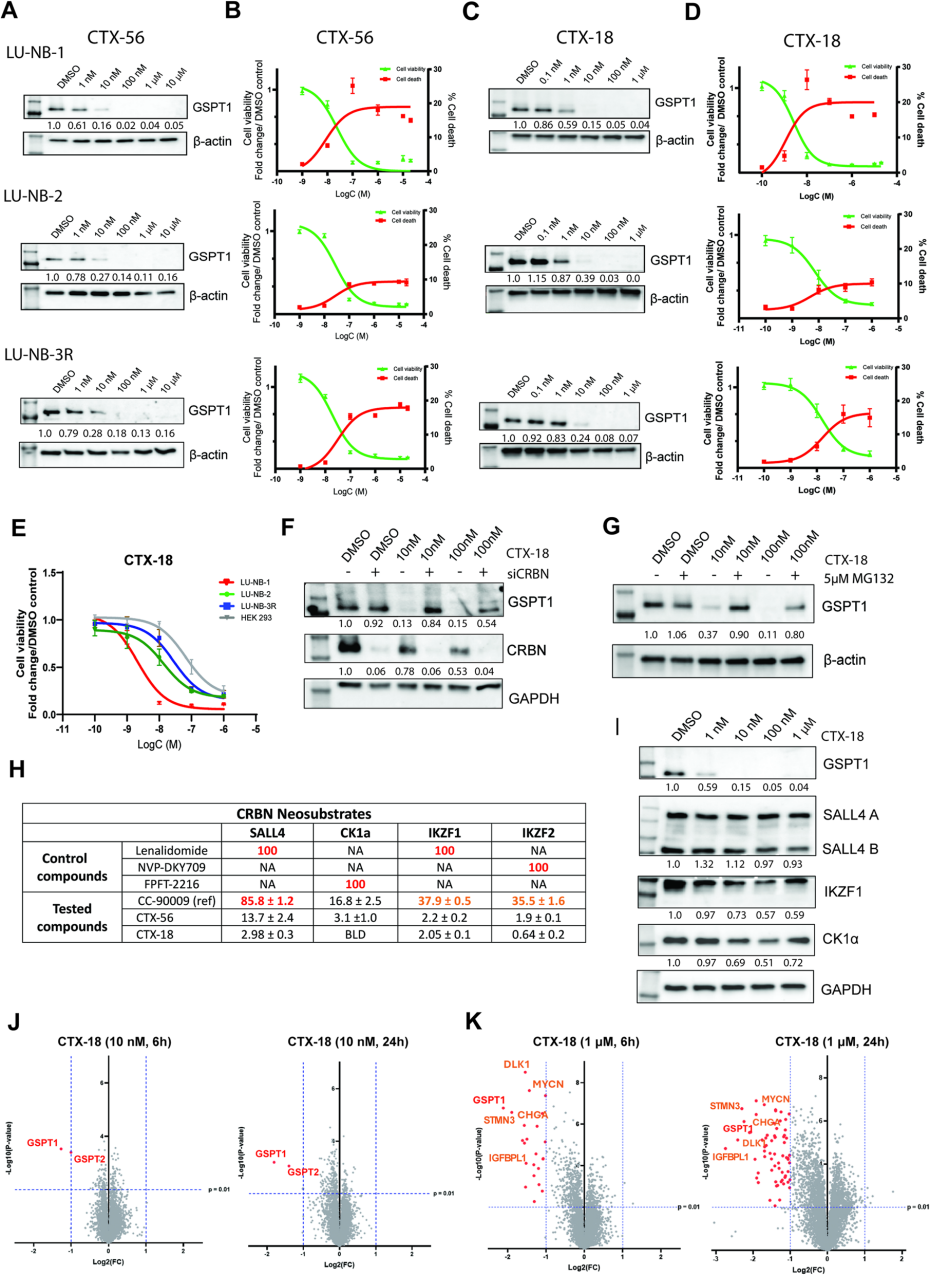
GSPT1 degradation in NB organoids. **A** Western blot analysis of GSPT1 protein levels in NB organoids (LU-NB-1, LU-NB-2, LU-NB-3R) after treatment with CTX-56 for 24 h. Quantification shows the fold change expression compared with DMSO control presented as the mean of 3 biological replicates; **B**) Cell viability and cell death in NB organoids following GSPT1 degradation with CTX-56. Organoids were treated for 72 h. 3 biological replicates (x3 technical replicates) were performed for each model; **C**) Western blot analysis of GSPT1 protein levels in NB organoids after treatment with CTX-18 for 24 h. Quantification shows the fold change expression compared with DMSO control presented as the mean of 3 biological replicates; **D**) Cell viability and cell death in NB organoids following GSPT1 degradation with CTX-18. Organoids were treated for 72 h. 3 biological replicates (x3 technical replicates) were performed for each model; **E**) Cell viability of NB organoids LU-NB-1, LU-NB-2, LU-NB-3R, and HEK293 cells after CTX-18 treatment for 72 h. 3 biological replicates were performed; **F**) Western blot analysis of GSPT1 levels following co-treatment with CTX-18 and siCRBN in the LU-NB-1 model. Quantification shows the fold-change expression compared with DMSO control presented as the mean of 3 biological replicates; **G**) Western blot analysis of GSPT1 levels following co-treatment with CTX-18 and proteasome inhibitor MG132 in LU-NB-1 organoids. Cells were pre-treated with 5 µM MG132 for 1 h, followed by 6-hour co-treatment with CTX-18 at 10 nM and 100 nM. Quantification shows the fold change in expression compared with DMSO control presented as the mean of 3 biological replicates; **H**) Analysis of compound selectivity in the AlphaLISA-based ternary complex formation assay. Luminescence observed for the neosubstrate-CRBN system at 10 µM of tested MGs (CC-90009, CTX-56, CTX-18) was normalized to signal observed for the reference compound at the same concentration. The normalized response is expressed as a percentage of the reference value (mean ± SD): 0–25% (green), 26–75% (orange), 76–100% (red). NA – not analyzed; BLD – below limit of detection; **I**) Western blot analysis of CRBN-neosubstrate levels in LU-NB-1 organoids treated with CTX-18 for 24 h. Quantification shows the fold change in expression compared with DMSO control presented as the mean of 3 biological replicates. Samples were run on 2 separate gels, and appropriate loading controls were used for each gel (representative loading control is shown); **J**) Proteomic analysis showing downregulation of proteins in LU-NB-1 organoids after treatment with 10 nM CTX-18 for 6 h (left) or 24 h (right). ~6770 proteins were detected; downregulated proteins are marked in red. A *p*-value < 0.01 was considered significant. The data show the results from 4 biological replicates; **K**) Proteomic analysis showing downregulation of proteins in LU-NB-1 organoids after treatment with 1 µM CTX-18 for 6 h (left) or 24 h (right). ~6770 proteins were detected; downregulated proteins are marked in red, downregulated NB-related proteins are marked in orange. A *p*-value < 0.01 was considered significant. The data show the results from 4 biological replicates

We next tested CTX-56 and CTX-18 in *MYCN*-amplified NB organoids. Both compounds induced dose-dependent degradation of GSPT1 protein, with high efficacy and nanomolar DC_50_ values (Fig. 4A, C, Supplementary Material 2). GSPT1 degradation reduced NB cell viability with IC_50_ values from 3 to 30 nM after 72 h of treatment (Fig. 4B, D), suggesting that GSPT1 plays a fundamental role in NB cell growth. Additionally, there was a substantial increase in NB cell death in the treated organoids (Fig. 4B, D). The effects of both tested MGs were comparable to the reference compound CC-90009 (Supplementary Fig. 3A, B, Supplementary Material 2). The impact of long-term GSPT1 degradation was also evaluated and, while the effect on cell viability was comparable to 72 h of treatment, NB cell death was significantly higher after a week of treatment (Supplementary Fig. 3C). The detailed DC_50_, IC_50_, and I_Max_ values for each model are shown in Supplementary Fig. 3D. Additionally, the effect of GSPT1 degradation was analyzed in HEK293 cells, where sensitivity to GSPT1 degradation was decreased after 72 h of treatment, especially when compared to the more aggressive LU-NB-1 model (LU-NB-1 IC_50_ ~ 2 nM, HEK293 IC_50_ ~ 130 nM, Fig. 4E).

To validate the dependency of GSPT1 degradation on CRBN activity, *CRBN* was knocked down transiently in NB organoids. Reduced GSPT1 degradation, and consequently diminished MG activity, was observed in cells lacking CRBN compared with controls, indicating that CRBN recruitment is crucial for GSPT1 degradation and its mediated effects (Fig. 4F, Supplementary Fig. 3E, Supplementary Material 2). Similarly, co-treatment of the organoids with GSPT1-targeting MG and the proteasome inhibitor MG132 also abrogated GSPT1 degradation, confirming that proteasome recruitment mediates GSPT1 degradation with the tested compounds (Fig. 4G, Supplementary Fig. 3F, Supplementary Material 2).

An important parameter in MG degrader development is their potential for off-target activity. We therefore performed biophysical and cellular assays to examine whether the strong anti-proliferative effects were due to selective GSPT1 degradation. The AlphaLISA-based ternary complex formation assay demonstrated a lack of binding of the tested MGs to the most common cereblon neosubstrates (CK1α, SALL4, or IKZF1), suggesting high specificity towards GSPT1 (Fig. 4H). In contrast, the reference compound CC-90009 strongly bound SALL4 and showed medium interactions with IKZF1 and IKZF2 (Fig. 4H). CC-90009-mediated SALL4 degradation was also confirmed in vitro in the LU-NB-1 model (Supplementary Fig. 4A, Supplementary Material 3). Moreover, the efficacy of the tested MGs was abrogated in cells expressing mutated *GSPT1* (G575N), which prevents its recruitment to CRBN, compared to WT *GSPT1*, with 1000-fold difference in IC_50_ values (Supplementary Fig. 4B, C). Furthermore, treatment of NB organoids with increasing concentrations of CTX-18 induced selective GSPT1 degradation with minimal effects on CK1α, SALL4, or IKZF1 levels (Fig. 4I, Supplementary Fig. 4D, Supplementary Material 3).

Global proteomic analysis showed relatively clean selectivity profiles towards GSPT1/GSPT2 after short (6 h) and long term (24 h) treatment with 10 nM CTX-18 in a query of ~ 6.5k proteins (Fig. 4J, Supplementary Fig. 4E). Treatment of the cells with a high dose (1 µM) downregulated numerous proteins. However, only a few of these were identified and downregulated (> 2 fold) in both tested organoid models after 6 h: stathmin-3 (STMN3), MYCN, amyloid-β precursor protein (APP), chromogranin-A (CHGA), synaptotagmin-11 (SYT11), and Golgi membrane protein 1 (GOLM1) (Table S1_Proteomics). Several of the affected proteins are known to be involved in NB progression, including MYCN, CHGA, DLK1 (protein delta homolog 1), and STMN3 (Fig. 4K, Supplementary Fig. 4F). The MG-mediated degradability of 25 potential CRBN off-targets was assessed using a recently described bioinformatics approach [29], with tool validity first confirmed by verifying the presence of the G-loop motif in GSPT1, GSPT2, SALL4, CK1α, and IKZF1. Only 5 proteins (APP, RNF138, SYT4, PRAME, and TRIB2) contained an exposed G-loop, suggesting the observed downregulation was likely secondary to GSPT1 degradation, a conclusion supported by the higher number of proteins downregulated after 24 h (Fig. 4K, Supplementary Fig. 4F, Table S1_Proteomics).

The lack of significant degradation of SALL4, CK1α, and IKZF1 in proteomic experiments correlated with WB-based degradation measurements for these proteins (Table S1_Proteomics, Fig. 4I). No other known CRBN off-targets were detected in LU-NB-1. Only IKZF2 was downregulated in LU-NB-2 following prolonged treatment with MG at a concentration exceeding the DC_50_ by 1000-fold (Supplementary Fig. 4F). The full list of downregulated proteins is listed in Supplementary Material 4 and 5.

Thus, treatment of *MYCN*-amplified NB organoids with MGs resulted in: (1) proteasome-dependent GSPT1 degradation with minimal effects on CK1α, SALL4, or IKZF1; (2) higher GSPT1-specificity compared to the reference compound CC-90009; and (3) impaired cell viability and increased cell death.

### GSPT1 degradation induces caspase-mediated apoptosis in NB

**Fig. 5:**
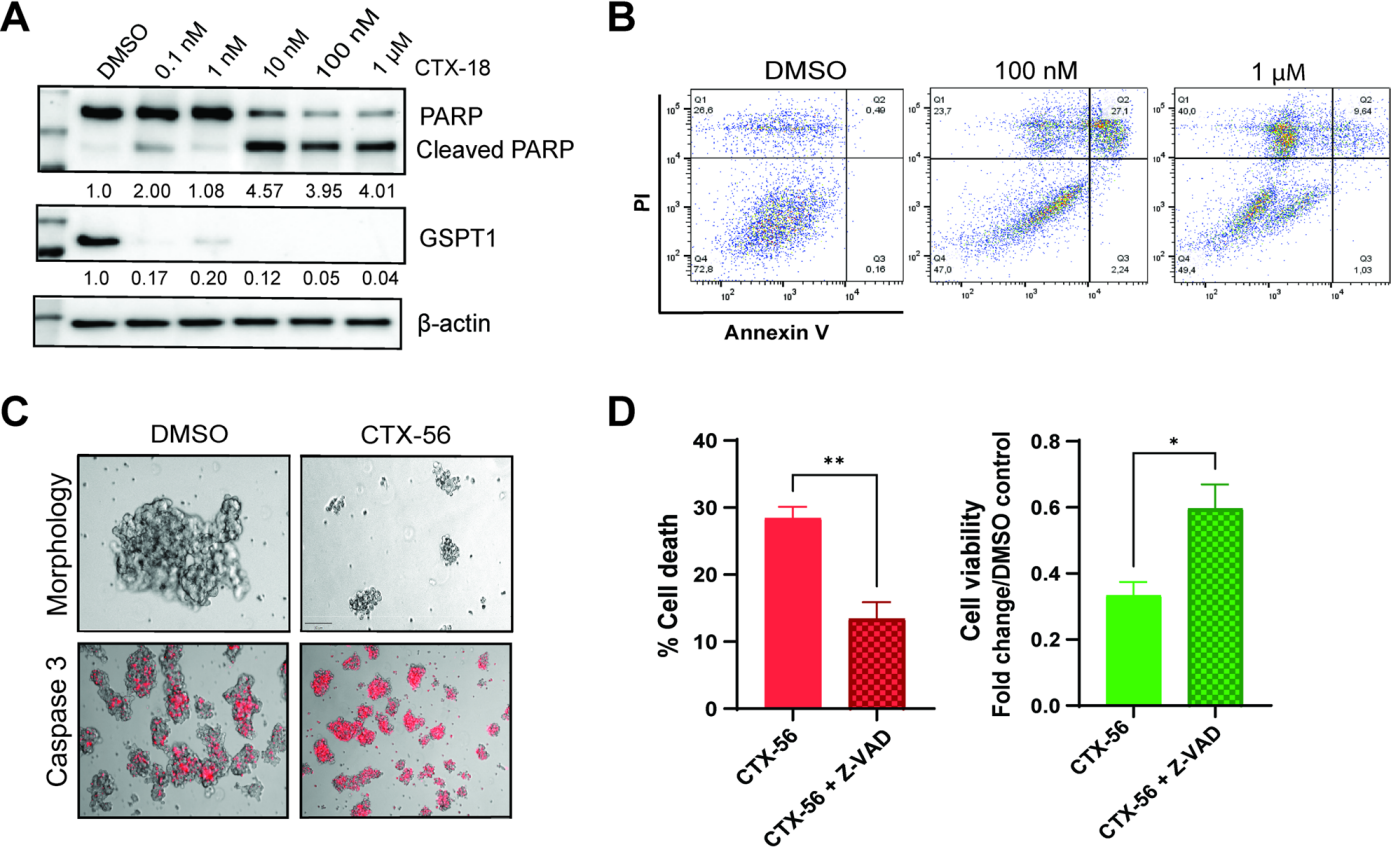
GSPT1 degradation induces caspase-mediated apoptosis in NB organoids. **A** Western blot analysis of PARP/cleaved PARP levels in LU-NB-1 organoids following 24 h treatment with CTX-18. Quantification shows the cleaved PARP/PARP to actin ratio presented as fold change compared with DMSO control (mean of 3 independent experiments); **B**) Flow cytometry analysis of Annexin V/PI-positive LU-NB-1 cells 48 h after treatment with CTX-56 at 100 nM and 1 µM concentrations; **C**) Morphological changes (brightfield imaging, upper panel) and activity of caspase 3 (brightfield + fluorescence, lower panel) in LU-NB-1 organoids 48 h after treatment with 1 µM CTX-56. 50 μm (upper panel) and 100 μm (lower panel) scale were used; **D**) Cell death and cell viability of LU-NB-1 organoids after co-treatment with 100 nM CTX-56 and the pan-caspase inhibitor Z-VAD, compared with CTX-56 treatment alone. 4 biological replicates were performed. t-test with Welch correction was used for statistical analysis

Considering the strong effects of GSPT1 degradation on increasing NB cell death, we examined the impact of GSPT1 degradation on NB apoptosis. GSPT1 degradation enhanced levels of cleaved PARP (Fig. 5A, Supplementary Material 3) and elicited a significant increase in the proportion of late apoptotic cells 48 h after treatment, as shown by Annexin V/PI staining (Fig. 5B, Supplementary Fig. 4G, H), especially in the LU-NB-1 model. In addition, GSPT1 degradation significantly altered NB organoid morphology, with organoid defragmentation, formation of apoptotic bodies, and increased caspase 3 activity (48 h post treatment compared with DMSO controls; Fig. 5C, Supplementary Fig. 4I). In addition, co-treatment of LU-NB-1 organoids with a GSPT1 degrader and the pan-caspase inhibitor Z-VAD (OMe)-FMK reduced NB cell death (*p* = 0.0037) and increased NB cell viability (*p* = 0.0273) compared with GSPT1 degradation alone (Fig. 5D). This co-treatment did not produce significant effects in two other NB models (Supplementary Fig. 4J).

Taken together, our findings indicate that GSPT1 degradation induces apoptosis in *MYCN*-amplified NB.

### Degradation of GSPT1 shows therapeutic effects in NB PDX models in vivo

Given the strong efficacy of GSPT1-targeting MGs in vitro, we evaluated the therapeutic efficacy of GSPT1 degradation in vivo by testing CTX-18 in two chemoresistant *MYCN*-amplified NB PDX models: PDX1 and PDX3R. Drug metabolism and pharmacokinetics (DMPK) characterization showed that the compound exhibited good plasma stability in mice and humans and a satisfactory pharmacokinetics profile with ~ 50% bioavailability when administered systemically via oral gavage (Supplementary Fig. 5A).

**Fig. 6:**
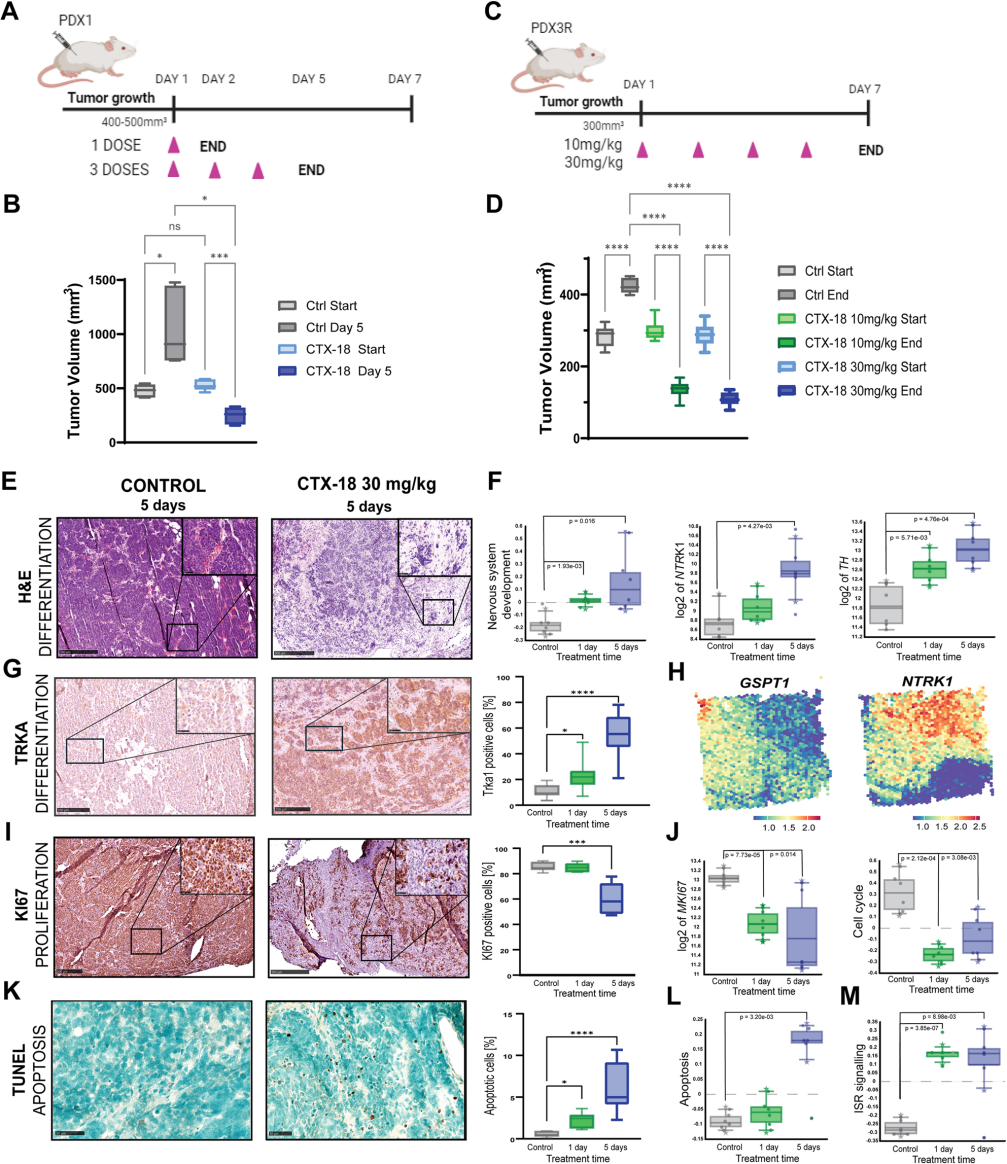
GSPT1 degradation in a *MYCN*-amplified NB PDX model in vivo. **A** Schematic overview of the short-term in vivo study (NB PDX1). Mice were treated for 24 h (1 dose, *n* = 6) or 5 days (3 doses administered on days 1, 3, and 4; *n* = 6) with 30 mg/kg CTX-18 (p.o.). Control mice (*n* = 6) were treated with vehicle for 5 days; **B**) Tumor volumes of NB PDX1 tumors following 5 days of treatment with 30 mg/kg CTX-18 or vehicle, tested with one-way ANOVA with multiple comparison correction for statistical analysis; **C**) Schematic overview of the short-term in vivo study (NB PDX3R). Mice were treated with 10 mg/kg (*n* = 6) or 30 mg/kg (*n* = 6) CTX-18 4 times over 7 days (on days 1, 2, 4, and 6), while control mice (*n* = 6) were treated with vehicle for 7 days; **D**) Tumor volumes of PDX3R tumors following the 7 day treatment with vehicle, 10 mg/kg CTX-18, or 30 mg/kg CTX-18. One-way ANOVA with multiple comparison correction was used for statistical analysis; **E**) H&E staining of controls and CTX-18-treated PDX1 tumors after 5 days of treatment (scale bars: 500 and 50 μm); **F**) Expression levels of genes associated with nervous system development and differentiation markers *NTRK1* and *TH* in control and treated PDX1 tumors. Statistical significance was assessed using Welch’s ANOVA; **G**) Representative immunohistochemistry staining and quantification of TrkA levels in controls and CTX-18-treated PDX1 tumors (scale bars: 250 and 50 μm). One-way ANOVA was used for statistical analysis, and five tumors were analyzed per condition; **H**) Spatial expression patterns of *GSPT1* and *NTRK1* in a *MYCN*-amplified NB patient tumor [26]; **I**) Representative IHC staining and quantification of Ki67 expression in control and CTX-18-treated PDX1 tumors: (scale bars: 500 and 50 μm). One-way ANOVA was used for statistical analysis, five tumors were analyzed per condition; **J**) Expression levels of *MKI67* and cell cycle-associated genes in control and treated PDX1 tumors. Statistical significance was assessed using Welch’s ANOVA; **K**) TUNEL staining and quantification in control and CTX-18-treated PDX1 tumors. Representative images are shown (scale bar: 50 μm). One-way ANOVA was used for statistical analysis, four tumors were analyzed per condition; **L**) Expression levels of apoptosis-related genes in control and treated PDX1 tumors. Statistical significance was assessed using Welch’s ANOVA; **M**) Expression levels of ISR-related genes in control and CTX-18-treated PDX1 tumors. Statistical significance was assessed using Welch’s ANOVA

NSG mice bearing PDX1 tumors were treated with 30 mg/kg CTX-18 for either 1 day (*n* = 6, 1 administration) or 5 days (*n* = 6, 3 administrations) or with vehicle (*n* = 6, 5 days, 3 administrations) once tumors reached ~ 400–500 mm^3^ (Fig. 6A). Short-term treatment of PDX1 mice with CTX-18 significantly decreased tumor size (CTX-18 start vs. CTX-18 day 5, *p* = 0.0002, Ctr day 5 vs. CTX-18 day 5, *p* = 0.0104) after 3 administrations (Fig. 6B, Suppl Fig. 5B). Similarly, treatment of mice bearing PDX3R tumors decreased tumor volume after 7 days (CTX-18 10 mg/kg and 30 mg/kg, *p* < 0.0001, Fig. 6C, D).

Western blot analysis demonstrated almost complete GSPT1 degradation in the treated PDX1 tumors after both treatment periods (1 day, 5 days, Supplementary Fig. 5C), confirming strong pharmacodynamic activity of CTX-18. Histopathological analysis of treated PDX1 tumors showed morphological changes including enrichment of neurofibrillary matrix and Homer-Wright rosettes, suggesting NB differentiation (Fig. 6E, Supplementary Fig. 5D). Similarly, transcriptome analysis of treated tumors revealed increased expression of the KEGG gene signature related to nervous system development and differentiation-related markers including *NTRK1* or *TH* (Fig. 6F). Protein TrkA expression was also enhanced in the treated tumors (5 days vs. ctrl *p* < 0.0001, 1 day vs. ctrl *p* = 0.0197; Fig. 6G, Supplementary Fig. 5E). Consistently, spatial transcriptomic analysis of NB patient tumors [26] demonstrated lower GSPT1 expression in tumor regions with a prevalence of more differentiated late NB-like cells (Tumor1) vs. the undifferentiated tumor regions (Tumor2) (Fig. 6H, Supplementary Fig. 5F, [30]). This was further confirmed by the inverse expression of *GSPT1* and *NTRK1*, with low *GSPT1* expression in more differentiated *NTRK1*-high compartments (Fig. 6H, Supplementary Fig. 5F). IHC analysis of PDX1 tumors showed a decrease in Ki67-positive cells (5 days vs. ctrl, *p* = 0.0009), indicating a reduction in proliferating NB cells following GSPT1 degradation (Fig. 6I, Supplementary Fig. 5G). Transcriptomic analysis confirmed reduced expression of *MKI67* and cell cycle-related genes (KEGG database, Fig. 6J) in tumors with GSPT1 degradation. In addition, treated tumors showed increased formation of apoptotic bodies and enhanced TUNEL staining, with an increase in the proportion of apoptotic cells (0.5% of apoptotic cells in ctrl tumors vs. 5.5% in 5-day-treated tumors, *p* < 0.0001; Fig. 6K, Supplementary Fig. 5H). Similarly, transcriptome analysis revealed augmented expression of apoptosis-related genes (Fig. 6L). Treated tumors also showed an increased integrated stress response (ISR)-associated gene signature, suggesting activation of ISR in response to GSPT1 degradation (Fig. 6M).

Together, short-term GSPT1 degradation in *MYCN*-amplified NB PDX models resulted in a strong antitumorigenic response via decreased NB cell proliferation, increased apoptosis, and induction of cell differentiation.

### Degradation of GSPT1 reduces MYCN activity in vivo

**Fig. 7:**
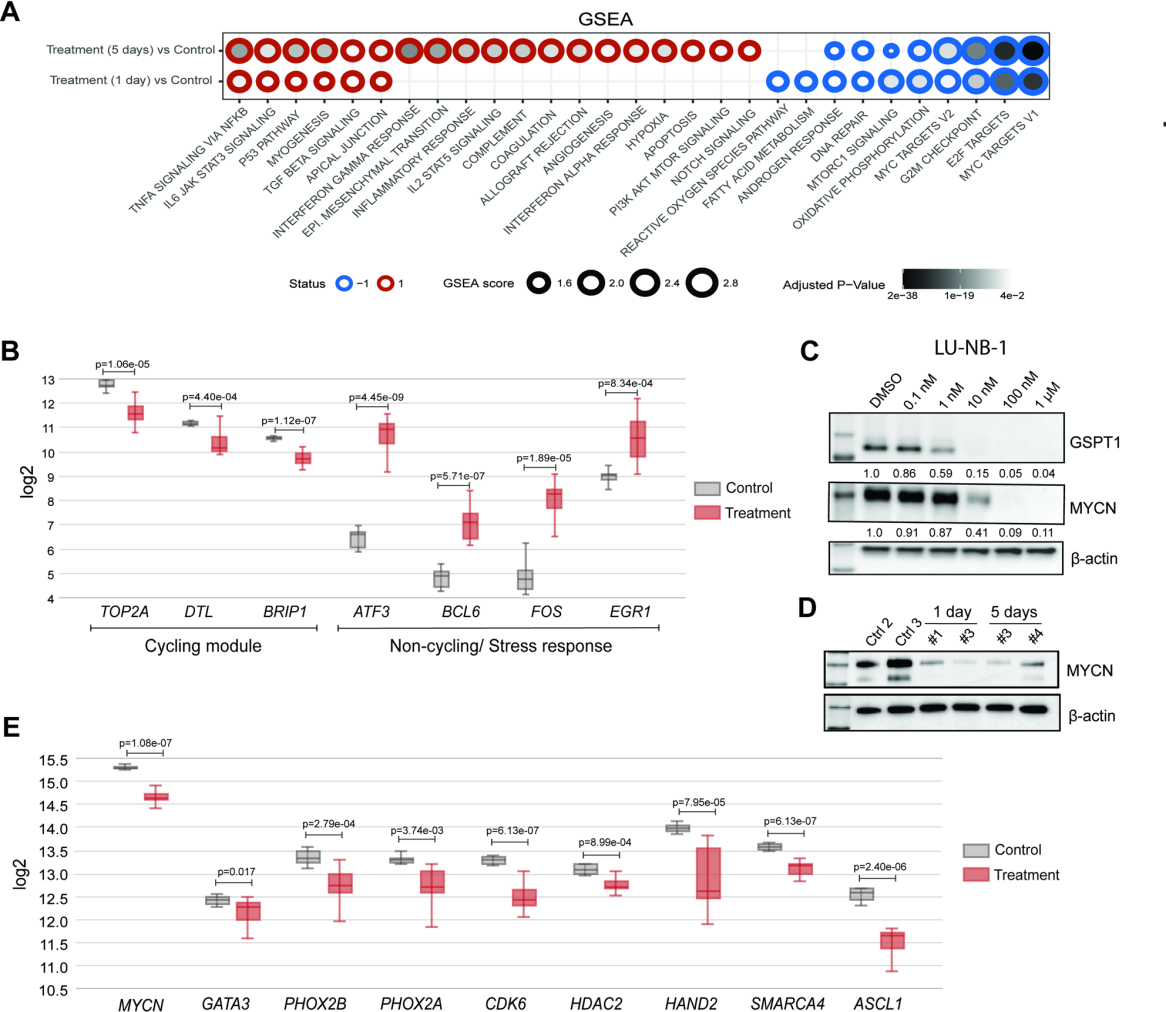
GSPT1 degradation leads to MYCN suppression in chemoresistant *MYCN*-amplified neuroblastoma. **A** Gene Set Enrichment Analysis (GSEA) of PDX1 tumors showing pathways upregulated (red) and downregulated (blue) following treatment with CTX-18 (30 mg/kg for 1 or 5 days) relative to control tumors. Pathways with *p* < 0.05 were considered statistically significant; **B**) Changes in mRNA expression of genes associated to cycling persisters, non-cycling cells, and stress response-related genes [31] following treatment with CTX-18 in vivo. Statistical significance was assessed using a two-tailed *t*-test; **C**) Western blot analysis of MYCN protein levels in LU-NB-1 organoids following CTX-18 treatment for 24 h. The quantification is a mean of 3 biological replicates presented as fold-change levels compared with DMSO; **D**) Western blot analysis of MYCN protein levels in PDX1 tumors from mice treated with vehicle (Ctrl2, Ctrl3) or CTX-18 (30 mg/kg) for 1 day (1, 3) or 5 days (3, 4); **E**) mRNA levels of *MYCN* and *MYCN*-associated core regulatory circuitry genes in PDX1 tumors following treatment with CTX-18 in vivo (1 day and 5 days treatment combined). Statistical significance was assessed using a two-tailed *t*-test

Gene Set Enrichment Analysis (GSEA) of the PDX1 tumors revealed that GSPT1 degradation in vivo increased levels of gene signatures related to NFĸB signaling, IL6/JAK/STAT signaling, and interferon γ/α response (Fig. 7A), thus, a proinflammatory and enhanced immune response. Treatment also strongly downregulated E2F activity and MYC targets and disrupted pathways involved in DNA repair, G2M checkpoint control, and oxidative phosphorylation. Moreover, treated tumors were characterized by reduced expression of markers specific for NB cycling persister cells (Fig. 7B; *TOP2A*,* DTL*,* BRIP1*), which is associated with a better prognosis [31]. At the same time, there was a strong increase in expression of genes characterizing non-cycling/stress response-related genes (*ATF3*,* BCL6*,* FOS*,* EGR1*) in treated tumors, suggesting that GSPT1 degradation may preferentially target highly proliferating cells.

Considering the strong downregulation of MYC targets, we analyzed MYCN protein levels. Treatment of NB organoids with CTX-18 in vitro decreased MYCN abundance (Fig. 7C, Supplementary Fig. 5I, Supplementary Material 3). Similarly, WB analysis of tumors treated in vivo demonstrated a strong decrease in MYCN levels following both 1 and 5 days of treatment (Fig. 7D). Furthermore, transcriptomic analysis of the treated PDX1 tumors showed strong downregulation of *MYCN* mRNA levels and decreased expression of MYCN-associated super-enhancer genes, including *PHOX2B*,* PHOX2A*,* CDK6*,* HDAC2*,* HAND2*,* SMARCA4*, and *ASCL1* (Fig. 7E, [32, 33]). Significantly decreased expression was apparent after only 1 day of the treatment, suggesting that the observed effects were not entirely related to cell death (Supplementary Fig. 5J). There were no differences in *MYC* or *MYCL* gene expression after treatment (Supplementary Fig. 5K), suggesting that the observed effects are due to specific *MYCN* downregulation.

Thus, interference of translation termination by GSPT1 degradation in vivo leads to extensive transcriptomic changes including suppression of *MYCN* gene expression and its associated CRC genes as well as reduced MYCN protein levels.

### GSPT1 degradation leads to long-term survival in a chemoresistant HR-NB PDX model

**Fig. 8:**
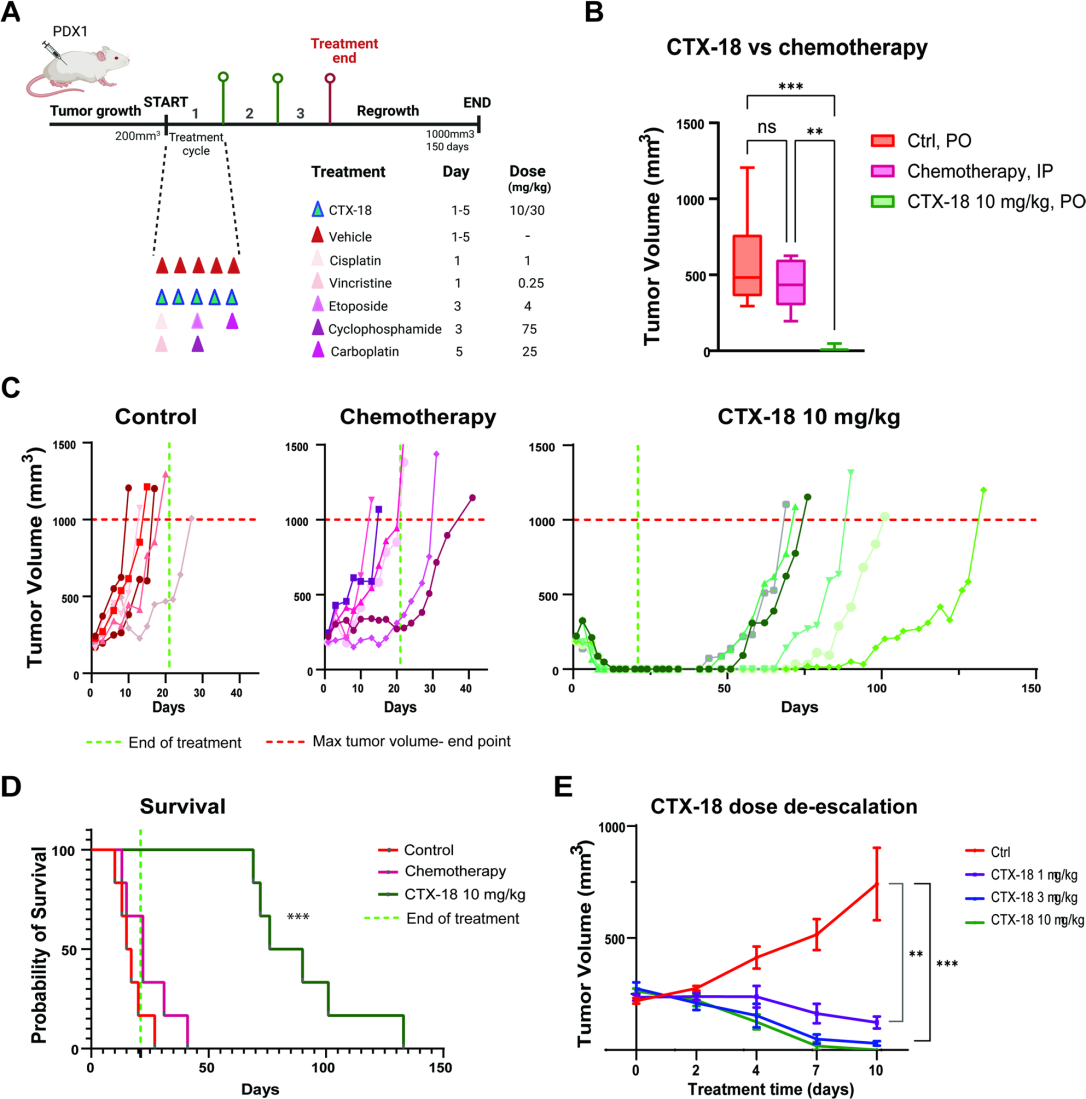
GSPT1-degradation prolongs survival of mice with chemoresistant *MYCN*-amplified PDX tumors. **A** Schematic overview of the experimental design of the long-term survival study in mice bearing PDX1 tumors. Two doses of CTX-18 (10 and 30 mg/kg, p.o.) were administered. A standard-of-care combination chemotherapy regimen was administered via i.p. injections as a control treatment. Treatment was given for 3 weekly cycles; **B**) Tumor volumes at day 10 of treatment with CTX-18 and chemotherapy (Tukey’s multiple comparison test of all groups). PO - oral administration, IP - intraperitoneal injection; **C**) Tumor volume growth curves for individual mice described in (A). Treatment groups: control (n = 6, red), chemotherapy (n = 6, purple), CTX-18 10 mg/kg (n = 6, green). The green line indicates end of treatment (21 days), the red line indicates the maximal tumor volume (euthanasia criteria); **D**) Kaplan-Meier survival curves showing survival of mice treated with CTX-18 at 10 mg/kg compared with control or standard-of-care chemotherapy. The green line indicates end of treatment. **E**) Tumor volume growth curves for PDX1 tumors in mice treated with vehicle or CTX-18 (1 mg/kg, 3 mg/kg, 10 mg/kg, n = 4 in treated groups, n = 5 in controls)

Considering the promising efficacy of short-term GSPT1 degradation in vivo, we examined long-term effects of GSPT1 targeting in a chemoresistant NB PDX model. NSG mice harboring ~ 200 mm^3^ PDX1 tumors (Supplementary Fig. 6A) were treated with CTX-18 or vehicle for three 1-week cycles (oral gavage, 5 days on and 2 days off). Treatment response was directly compared to standard-of-care COJEC induction chemotherapy (cisplatin, vincristine, etoposide, cyclophosphamide, carboplatin) [21] administered by intraperitoneal (i.p.) injections in weekly cycles (Fig. 8A).

The compound was well-tolerated, and no weight loss was observed in either of the CTX-18 treatment groups (Supplementary Fig. 6B). Chemotherapy-induced toxicity was observed in 1 of 6 treated mice. Treatment with CTX-18 (10 mg/kg) led to a striking and robust anti-tumor response, with complete tumor regression observed after only 1 week of treatment (day 10; 10 mg/kg CTX-18 vs. Ctrl, *p* = 0.0002; CTX-18 10 mg/kg vs. chemotherapy, *p* = 0.0037; Fig. 8B).

Analysis of the long-term response demonstrated that all control tumors and 4/6 chemotherapy-treated tumors displayed progressive disease, while 2 out of 6 chemotherapy-treated mice showed a partial response (Ctrl median survival: 16 days; chemotherapy median survival: 22 days; Fig. 8C). Treatment with 10 mg/kg CTX-18 led to complete tumor remission and increased survival (median survival time: 83 days, Fig. 8C), although 6/6 mice eventually relapsed (Fig. 8C). Survival analysis of treated mice demonstrated significantly increased survival following treatment with CTX-18 compared with COJEC chemotherapy (*p* = 0.0002; Fig. 8D). Notably, treatment with a higher dose of CTX-18 (30 mg/kg) led to long-term tumor regression in all mice, with 1/6 mice showing slow tumor regrowth after 120 days, and 5/6 mice had no signs of tumor growth at day 150, demonstrating complete and long-lasting tumor regression (Supplementary Fig. 6C). Importantly, no signs of metastases were noted in the cured mice (treatment with 30 mg/kg CTX-18), while 2/6 of the relapsed mice (10 mg/kg dose) developed liver metastases (Supplementary Fig. 6D). Given the potential risk of side-effects of GSPT1-degraders, we additionally performed an in vivo dose de-escalation study. Notably, CTX-18 retained anti-tumor efficacy at reduced doses of 1 mg/kg (*p* = 0.0033) and 3 mg/kg (*p* = 0.001) (PDX1 model, *n* = 4 per group; Fig. 8E).

Together, these results demonstrate strong antitumor efficacy for the GSPT1 degrader CTX-18 in an aggressive NB PDX model resistant to high-dose combination chemotherapy. The complete and long-lasting eradication of tumors by CTX-18 indicates the curative potential of GSPT1 degradation in *MYCN*-amplified NB.

## Discussion

The current standard-of-care for high-risk NB involves intensive multimodal high-dose chemotherapy. While often initially effective, this approach is associated with a high risk of severe and potentially lifelong toxicities. Moreover, many patients experience relapse with treatment-resistant disease and poor survival outcomes. Consequently, the development of therapies that demonstrate efficacy in the relapsed and refractory setting would represent a major advance in the treatment of high-risk NB.

TPD is a promising therapeutic strategy for malignancies driven by previously considered “undruggable” oncogenic drivers, including MYC(N) and other transcription factors. By enabling complete degradation of disease-driving proteins, TPD enhances potency, sustains target suppression, and has the potential to circumvent conventional resistance mechanisms [15].

Here we explored the therapeutic potential of GSPT1-degrading MGs to address the unmet need for new, effective therapies for patients with chemoresistant high-risk NB. We show that GSPT1 is frequently expressed in NB and its high expression is associated with *MYCN* amplification and poorer clinical outcomes. Selective degradation of GSPT1 impaired viability of *MYCN*-amplified NB organoids and induced apoptosis. In vivo treatment reduced NB cell proliferation, promoted apoptosis, and enhanced NB differentiation. Mechanistically, GSPT1 degradation decreased MYCN protein levels and downregulated MYCN-driven transcriptional programs. Notably, long-term treatment of a chemoresistant NB PDX model outperformed standard-of-care chemotherapy, eradicating tumors and increasing survival of mice. These findings highlight that inhibition of translation by GSPT1-degrading MGs represents a promising therapeutic strategy for chemoresistant *MYCN*-amplified NB.

Oncogenic stress and elevated protein synthesis can render cancer cells particularly sensitive to disruptions in the translational machinery [34]. This vulnerability appears to be especially pronounced in MYC(N)-driven tumors. Our findings suggest that *MYCN*-amplified NB tumors are dependent on the high rate and fidelity of translation, highlighting a therapeutic vulnerability that can be exploited by GSPT1 degradation.

We and others have previously shown that chemotherapy can induce suppression of MYCN activity in non/slowly proliferating NB cells, even in the context of *MYCN* amplification [21, 31]. This is consistent with the concept of a chemotherapy-induced embryonic diapause-like state, where cancer cells suppress MYC activity to reduce redox stress and apoptotic priming to persist through therapy [35]. Consistent with these findings, we show that GSPT1-degradation decreased cell proliferation and suppressed MYCN activity at the transcriptional and protein levels in *MYCN*-amplified NB. These findings suggest that MYCN suppression may be a broader mechanism used by NB to adapt and survive under stressful conditions by shifting from a proliferative state towards a more quiescent and resistant phenotype.

GSPT1 expression in healthy tissues suggests the need for careful evaluation of the clinical safety of GSPT1-degraders. CC-885 (Celgene) was the first MG degrader of GSPT1, but it suffered from numerous off-target effects and hence the possibility of adverse events. Its follow-up compound, CC-90009, has shown higher selectivity and lower degradation potential of relevant off-targets, and it has been evaluated in a phase 1 trial for patients with AML and relapsing/remitting AML [36, 37]. Nevertheless, some grade 3 and 4 side effects were still observed, including infections (47%), hypocalcemia (22%), and hypertension (13%) [38]. Over the years, newer generations of degraders have advanced to clinical trials (e.g. BTX-1188/Biotheryx [39], MRT-2359/Monte Rosa Therapeutics [40], ABS-752/Captor Therapeutics) [41] and preliminary data, such as for MRT-2359 in a phase 1/2 study, showed no appreciable grade 3 and 4 toxicities at doses selected for further evaluation in a phase 2 trial [42].

A subtle, yet functionally significant, difference in the CRBN sequence between humans and mice impairs the recruitment of neosubstrates in the murine system. As a result, in vivo toxicity assessment in NSG mice does not capture potential human-specific on-target toxicity effects. However, the degradation profile of CTX-18 in human NB organoids shows high specificity for GSPT1/GSPT2, characterized by the critical absence of degradation of frequently observed CRBN off-targets, including SALL4, CK1α, and IKZF1 [43]. Since IKZF1 degradation by CRBN MGs induces thrombocytopenia and neutropenia [44, 45], avoiding its direct downregulation is expected to increase degrader safety.

Our analysis of a large clinical datasets revealed that *CRBN* mRNA expression is relatively low in non-malignant tissues compared with NB tumor cells. This differential expression could suggest that CRBN-mediated degradation of GSPT1 may be more pronounced in NB cells, potentially offering a therapeutic window for MGs that minimizes effects on healthy tissues. In addition, preferential activity of GSPT1 degraders in cells with high NMYC and LMYC expression [46] suggests that MYC(N)-addicted cancer cells, such as NB, might be more susceptible to GSPT1 degradation while sparing normal cells.

In our study, GSPT1 degradation led to tumor remission in mice with aggressive chemoresistant NB PDXs; however, it remains to be determined whether comparable therapeutic doses can be achieved in children given the potential on-target toxicities noted above. Preliminary findings from a phase 1/2 clinical trial of the selective GSPT1 degrader MRT-2359 show prolonged clinical efficacy at clinically feasible doses and schedules in patients with castration-resistant prostate cancer resistant to androgen receptor therapy [42, 47]. These data indicate that prolonged, intermittent dosing regimens may mitigate the risk of adverse effects while preserving therapeutic efficacy.

Future work should focus on developing GSPT1-degrading MGs with increased specificity towards NB cells and adjusting treatment regimens to increase the therapeutic window and reduce the risk of side effects. Development of GSPT1 degraders with E3 ligases specific for NB cells might yield higher specific activity against tumor cells and fewer side-effects. Such an approach has been evaluated in two studies using DCAF15 as an E3 ligase recruited to degrade RBM39 for NB [48, 49]. Both studies demonstrated that DCAF15 is highly expressed in NB compared to other solid tumors and that NB was partially dependent on DCAF15 activity. A wider repertoire of E3 ligases could potentially increase the safety and clinical value of this class of compounds. Alternatively, MGs in the form of prodrugs or MG-antibody conjugates, e.g., ORM-6151/Orum Therapeutics [50], could increase efficacy and specificity.

## Conclusions

Here we show that GSPT1 degradation is effective in preclinical models of *MYCN*-amplified NB. Targeting the translational machinery and the integrated stress response may provide an orthogonal approach to chemotherapy and targeted therapies (e.g., ALK inhibitors). This strategy could enable combinatorial treatments that target multiple mechanisms of action in NB, potentially reducing the risk of resistance development. Although potential toxicities will require careful evaluation, our findings suggest that GSPT1 degradation may also be effective in patients with relapsed and chemoresistant NB.

## Supplementary Information

Supplementary Material 1.

Supplementary Material 2.

Supplementary Material 3.

Supplementary Material 4.

Supplementary Material 5.

Supplementary Material 6: Supplementary methods and references.

Supplementary Material 7: Figures S1-S6 with captions.

Supplementary Material 8: Table S1_proteomics.

## Data Availability

RNA-seq data from this study is deposited to R2: Genomic Analysis and Visualization Platform (http://r2.amc.nl) under the name: PDX Neuroblastoma-TPD-2024_045 - 18 - DESeq2 - ensh38e110. The transcriptomic patient data that were analyzed in this study (Tumor Neuroblastoma - Kocak - 649 - custom - ag44kcwolf, Tumor Neuroblastoma - SEQC - 498 - RPM - seqcnb1) were obtained from publicly available datasets stored in R2. Single cell transcriptomic data analyzed in the study were obtained from Oscar Bedoya-Reina and Noah Bonine as Seurat Objects and is available upon request with the permission of those authors. All other data associated with this study are available upon reasonable request from the corresponding author.

## Abbreviations

ALK: Anaplastic lymphona kinase
BCA: Bicinchoninic acid assay
CHGA: Chromogranin A
CK1α: Casein Kinase 1 alpha
CRC: Core regulatory circuit
DAB: 3,3′-diaminobenzidine
DC_50_: Half maximal degradation concentration
DCs: Dendritic cells
DLK1: Delta-like non-canonical Notch ligand 1
DMPK: Drug Metabolism and Pharmacokinetics
DMSO: Dimethyl sulfoxide
FBS: Fetal bovine serum
FDA: Food and drug administration
GSEA: Gene set enrichment analysis
GSPT1: G1 to S phase transition protein 1
HRP: Horseradish peroxidase
HR-NB: High-risk neuroblastoma
IC_50_: Half maximal inhibitory concentration
IHC: Immunohistochemistry
IKZF1: IKAROS family zinc finger 1
IP: Intraperitoneal
JAK/STAT: Janus kinase/signal transducer and activator of transcription
MGs: Molecular glues
NSG: NOD scid gamma
PBS: Phosphate-buffered saline
PDX: Patient-derived xenograft
PO: Per os
PROTACs: Proteolysis targeting chimeras
RBCs: Red blood cells
RIPA: Radioimmunoprecipitation Assay
SNP: Single nucleotide polymorphism
STMN3: Stathmin-like 3
TEAB: Triethylammonium bicarbonate
TMA: Tissue microarray
TPD: Targeted protein degradation
