# Supplementary material for: GSPT1-specific protein degradation is effective in preclinical models of chemoresistant MYCN-amplified neuroblastoma
Source: J Exp Clin Cancer Res. 2026 Feb 6;45:58. doi: 10.1186/s13046-026-03647-0 (PMC12918055; doi:10.1186/s13046-026-03647-0)

Quantification to WBs shown in Fig 4A, 4C and Supplementary Fig 3A

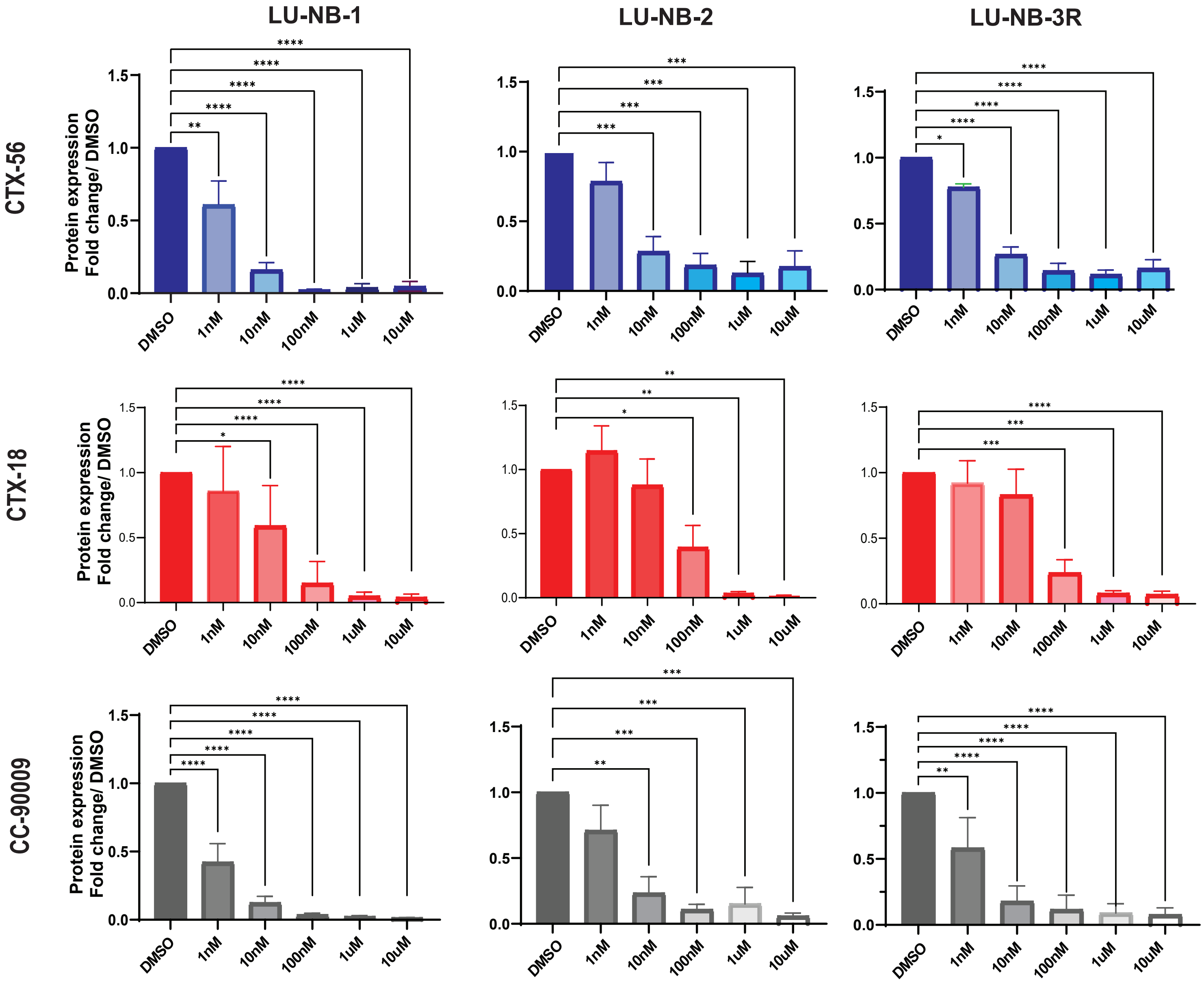

Quantification of WB shown in Fig 4F

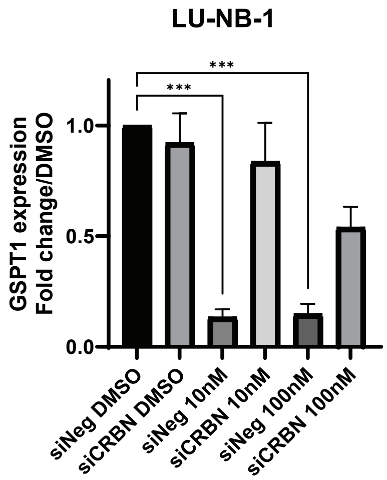

Quantification of WB shown in Fig 4G and Supplementary Fig 3F

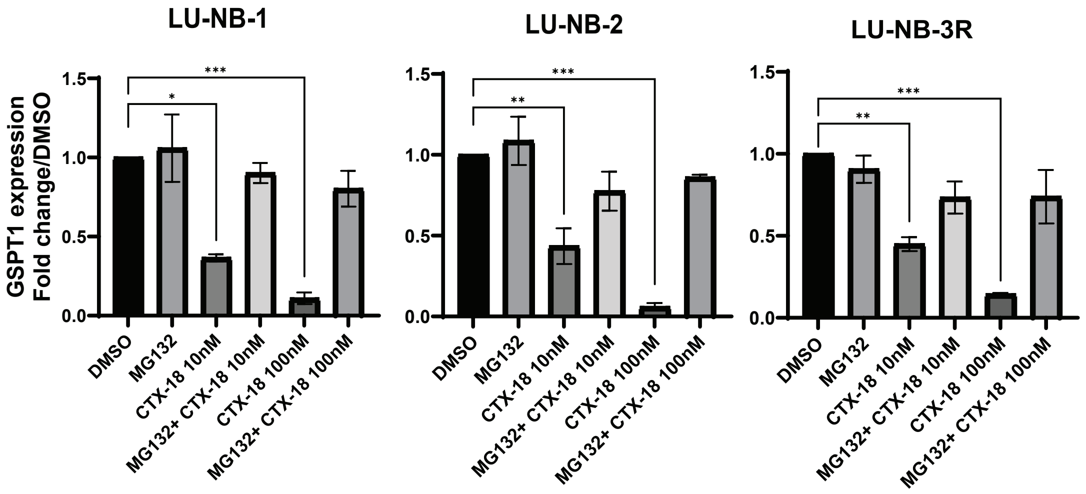

Supplement: Supplementary file 2 — Supplementary Material 2. [file 13046_2026_3647_MOESM2_ESM.pdf]
