# Supplementary material for: GSPT1-specific protein degradation is effective in preclinical models of chemoresistant MYCN-amplified neuroblastoma
Source: J Exp Clin Cancer Res. 2026 Feb 6;45:58. doi: 10.1186/s13046-026-03647-0 (PMC12918055; doi:10.1186/s13046-026-03647-0)

Quantification of WB shown in Fig 4I and Supplementary Fig 4G

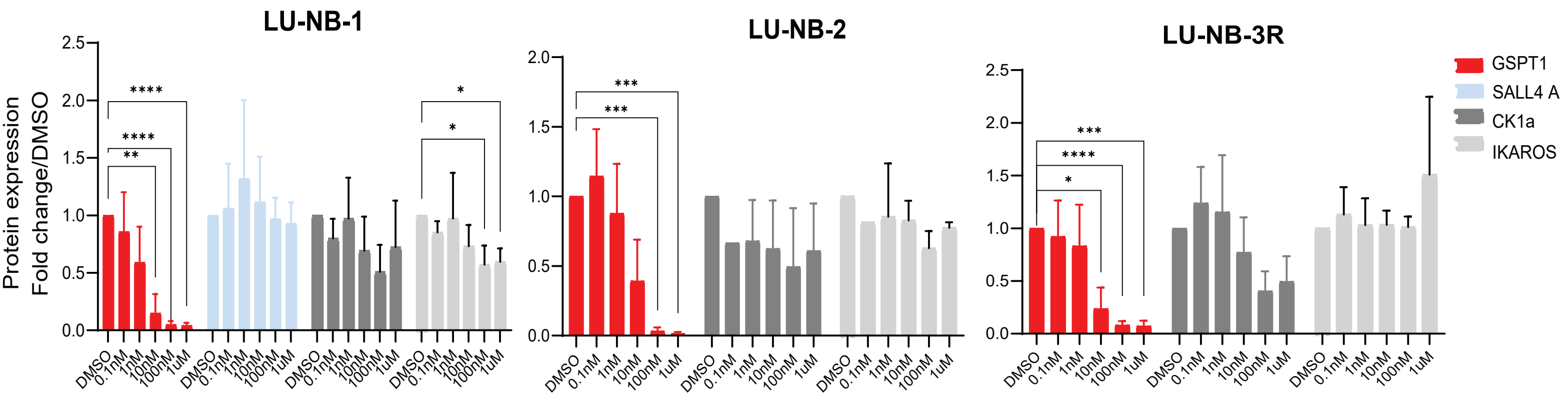

Quantification of WB shown in Fig 5A

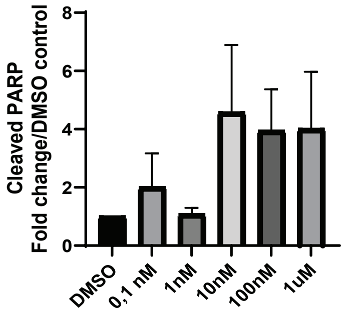

Quantification of WB shown in Fig 7C and Supplementary Fig 5I

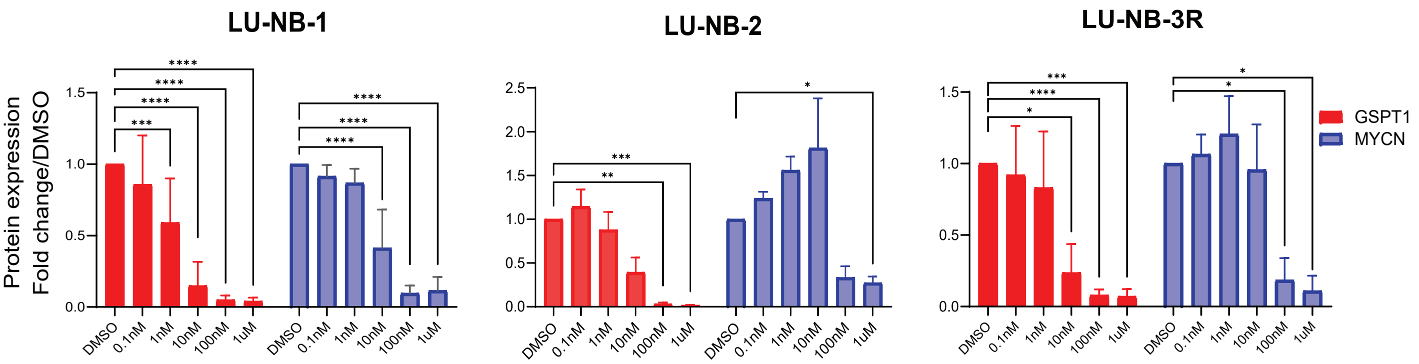

Supplement: Supplementary file 3 — Supplementary Material 3. [file 13046_2026_3647_MOESM3_ESM.pdf]
