# Supplementary material for: GSPT1-specific protein degradation is effective in preclinical models of chemoresistant MYCN-amplified neuroblastoma
Source: J Exp Clin Cancer Res. 2026 Feb 6;45:58. doi: 10.1186/s13046-026-03647-0 (PMC12918055; doi:10.1186/s13046-026-03647-0)
Supplement: Supplementary file 6 — Supplementary Material 6: Supplementary methods and references. [file 13046_2026_3647_MOESM6_ESM.docx]

**MATERIALS AND METHODS**

**Compound synthesis**

The compounds were prepared according to the published procedures CTX-56 (WO2022/144416, 2022), CTX-18 (WO2024/167423, 2024) or purchased from the commercial sources CC-90009 (MedKoo Biosciences, cat. no. 207005).

**HiBiT lytic assay and CTG viability assay**

GSPT1-HiBiT HEK293 cells were generated using the HEK293 parental cells (ATCC, cat. No 70016364) and the CRISPR/Cas9 system. HEK293 cells were transfected a plasmid carrying gRNA coding sequence targeting the C-terminus of GSPT1 (5’-> 3’: aattggaaaagttctgaaac) and ssODN template containing the HiBiT tag sequence with flanking homology sequences (5’->3’: ttaaaatttcatttctaggtaagaccattgcaattggaaaagttctgaaaGGGAGTTcTGGCGTGAGCGGCTGGCGGCTGTTCAAGAAGATTAGCaaactggttccagagaaagactaagcattttcttgatgaccctgcacaata) using the Neon Transfection System (Thermo Fisher Scientific). Single clones were verified by HiBiT Blotting (Promega) and genotyping. Cells were cultured in DMEM Glutamax medium, supplemented with 1% penicillin/streptomycinand 10% Fetal Bovine Serum (FBS) at 37°C, 5% CO_2_. For the Nano-Glo HiBiT assay as well as CellTiter-Glo assay (CTG assay), cells were seeded onto 384-well white solid bottom plate (a separate test plate for each assay) at 2 × 10^3^ cells/well in 40 μL growth medium volume using the Agilent BioTek MultiFlo FX Multimode Dispenser and incubated overnight. Cells were treated with the test compounds and vehicle only (DMSO) control using the Echo 555 Liquid Handler at given concentrations. Final DMSO concentration was kept constant at 0.25% v/v across the assay plates. After the indicated incubation time with the compounds luminescent signal detection was conducted with the Nano-Glo HiBiT Lytic Detection System (Promega, #N3050) or CellTiter-Glo Luminescent Cell Viability Assay (Promega, #G7571) according to manufacturer’s instructions using PHERAstar multimode plate reader (BMG LABTECH) for the Nano-Glo HiBiT Lytic assay and CLARIOstar multimode plate reader (BMG LABTECH) for CellTiter-Glo assay. Luminescence (RLU) values were normalized to the controls and reported absolute DC_50_ values were calculated using non-linear regression and appropriate equations. For the CTG assay with the NB Kelly cell line, cells were seeded onto a white 384-well solid bottom plate (Greiner, cat. No. 781080) at 2.5 × 10^3^ cells/well in 50 μL of growth medium volume using the MultiFlo FX dispenser and incubated overnight. The compound treatment, detection conditions (using a PHERAstar plate reader), and data analysis were identical to those used for the GSPT1-HiBiT HEK293 cells.

**NanoBit recruitment assay**

Human CRBN wild-type amino acid sequence was cloned into Promega pBiT2.1-N-[TK-SmBiT] vector and human GSPT1 wild-type and GSPT1(G575N) mutant amino acid sequences were cloned into Promega pBiT1.1-C-[TK-LgBiT] vectors following the Promega protocol (N2015, Technical Manual). The plasmids (with the ratio of 1 to 3 of SmBiT-tagged CRBN and LgBiT-tagged GSPT1 plasmids) were transfected into 293T cells using Lipofectamine 3000. Single-cell cloning of edited cell pool yielded 36 single clones, 3 of them carried the homozygous GSPT1(G575N) mutation identified by genotyping of the GSPT1 locus. Following transfection, the cells were treated with 1.19 μM NAE1 inhibitor MLN4924 for 24h and then with GSPT1 degraders for 10 min (DMSO was used as a control). Following the addition of the NanoBiT substrate (Promega, Nano-Glo Live Cell Reagent, cat.#N2011), luminescence was measured according to the manufacturer's protocol (N2015, Technical Manual).Raw luminescence signals from the DMSO wells were averaged (DMSO = 100%) and each compound treated well was normalized to DMSO (calculated as % of DMSO).Two biological replicates were performed.

**AlphaLISA assay**

AlphaLISA ternary complex formation assays were performed under reduced light. Reactions were conducted in PBS-DT buffer (20 mM phosphate buffer pH 7.4, 2.7 mM KCl, 137 mM NaCl, 1 mM DTT, 2% DMSO, supplemented with 0.1% Tween-20 for SALL4, IKZF1, and IKZF2 assays, or 0.01% Tween-20 for the CK1a assay). Composition of reaction mixtures for Zinc Finger proteins (SALL4, IKZF1, IKZF2): 400 nM Strep-tagged target, 100 nM CRBN/DDB1, 20 µg/mL Strep-Tactin Donor beads (PerkinElmer, AS106M), 20 µg/mL Anti-6xHis Acceptor beads (PerkinElmer, AL178M). Reaction mixture with CK1a: 20 nM target, 100 nM CRBN/DDB1, 20 µg/mL Streptavidin Donor beads (PerkinElmer, 6760002), 20 µg/mL Anti-6xHis Acceptor beads (PerkinElmer, AL178M). Protein-beads mixes were dispensed into 384-well plates (PerkinElmer, 6008350). Compounds (10µM, triplicates) and DMSO were added using Echo 555 liquid handler (Labcyte Inc.). Plates were sealed, mixed, centrifuged (10s, 1000g), incubated (30 min at RT) and read using an EnSpire plate reader (PerkinElmer). Luminescence for the tested and control compounds was corrected by subtracting the reading for wells containing DMSO (average value). Corrected luminescence was normalized to control compound (100%) for a particular target. CRBN/DDB1 and CK1a proteins were purified according to published protocols (1). Zinc Finger degrons of SALL4 (406-432 aa, UniProt: Q9UJQ4), IKFZ1 (141-174 aa, UniProt: Q13422) and IKZF2 (136-169 aa, UniProt: Q9UKS7) were cloned into pET28 vector encoding an N-terminal StrepTagII-AviTag-SUMO tag and expressed in *E. coli* BL21(DE3) cells in LB medium at 37°C. Cells were lysed using EmulsiFlex microfluidizer (Avestin) in 50 mM Tris/HCl (pH 8.0, 8°C), 300 mM NaCl, 5% glycerol, cOmplete EDTA-free inhibitor cocktail (Roche), 0.05% Triton X-100, 5 U/mL Viscolase (A&A Biotechnology), supplemented with 1 mM DTT. Recombinant proteins were captured from lysate with Streptactin XT 4flow resin (IBA Lifesciences) and eluted with buffer containing 5 mM biotin and 1mM DTT. Proteins were diluted to reduce NaCl concentration in sample (30 mM) and purified further with anion-exchange chromatography (HiTrap Q HP, Cytiva) utilizing NaCl gradient (30-500 mM). Size-exclusion chromatography polishing step was performed using HiLoad 16/600 Superdex 75 pg (Cytiva) in 50 mM Tris-HCl pH 8.0 (RT), 300 mM NaCl, 1 mM DTT buffer.

**Kinetic analysis of GSPT1 Ternary complex formation using SPR**

The affinity of GSPT1 (300-496 aa) protein to the CRBN-MG complex was determined by the SPR method. The biotinylated CRBN protein (CRBN (40-442 aa)-DDB1(1-395 aa)-Avi-DDB1(706-1140 aa)) was immobilized on a SA chip surface (Cytiva, BR100531) at approximately 1000 RU. GSPT1 and CRBN proteins were purified following published protocols (1), with modifications for the CRBN sequence and its enzymatic biotinylation. SPR experiments were performed using a Biacore 8K instrument (Cytiva). Both immobilization and interaction methods were conducted in an assay buffer (10 mM phosphate buffer pH 7.4, 2.7 mM KCl, 137 mM NaCl, 1 mM DTT, 0.01% Tween-20, 2% DMSO). Immobilization was performed at 25°C, and interaction measurements at 15°C. The chip surface was washed with assay buffer for 3 hours to achieve a stable baseline before measuring the GSPT1/MG-CRBN interaction. GSPT1 protein was serially diluted in assay buffer to obtain concentrations of 0.156, 0.312, 0.625, 1.25, 2.5, and 5 µM. GSPT1 binding was analyzed using the single-cycle kinetics method, where increasing concentrations of GSPT1 were injected (contact time: 60 s, flow rate: 30 µl/min). A dissociation phase followed the injection of the highest protein concentration (dissociation time: 600 s, flow rate: 30 µl/min). To determine the kinetics of GSPT1/MG-CRBN complex formation, sensorgrams for GSPT1 in mixture with 10 µM compound were corrected by subtracting sensorgrams from GSPT1-only injections. Solvent correction was included at the beginning and end of the experimental method. Collected data were processed and analyzed using Biacore Insight Evaluation Software, with fitting to a 1:1 binding model for single-cycle kinetics.

**RNA-seq Data Processing**

Raw sequencing data (FASTQ) underwent initial quality control using FastQC (v0.12.1) to assess overall read quality. Adapter trimming and removal of low-quality bases were performed using fastp (v0.24.0). Processed reads were then pseudoaligned to a human transcriptome reference, including both coding and non-coding transcripts from Ensembl release 112, using kallisto (v0.51.1) (2). Summary statistics and module metrics from the preprocessing steps were aggregated using MultiQC (v1.28).

Transcript-level abundance estimates from kallisto were imported into R for downstream analysis using tximport (v1.32.0) (3). Gene-level quality control and differential expression analysis followed the standard DESeq2 (v1.44.0) workflow, incorporating gene annotation information via biomaRt (v1.26.1). Log fold change shrinkage was applied using the apeglm method (v1.26.1) to stabilize variance estimates and improve interpretability of results.

**Supplementary References**

1. Przytulski K, Glaza P, Brach K, Sagan M, Statkiewicz G, Klajn J, et al. Comparative analysis of biophysical methods for monitoring protein proximity induction in the development of small molecule degraders. Biochim Biophys Acta Gen Subj. 2023;1867(9):130398.

2. Bray NL, Pimentel H, Melsted P, Pachter L. Near-optimal probabilistic RNA-seq quantification. Nat Biotechnol. 2016;34(5):525-7.

3. Soneson C, Love MI, Robinson MD. Differential analyses for RNA-seq: transcript-level estimates improve gene-level inferences. F1000Res. 2015;4:1521.
