# Supplementary material for: GSPT1-specific protein degradation is effective in preclinical models of chemoresistant MYCN-amplified neuroblastoma
Source: J Exp Clin Cancer Res. 2026 Feb 6;45:58. doi: 10.1186/s13046-026-03647-0 (PMC12918055; doi:10.1186/s13046-026-03647-0)
Supplement: Supplementary file 7 — Supplementary Material 7: Figures S1-S6 with captions. [file 13046_2026_3647_MOESM7_ESM.docx]

**SUPPLEMENTARY FIGURES**

**Supplementary Fig S1**


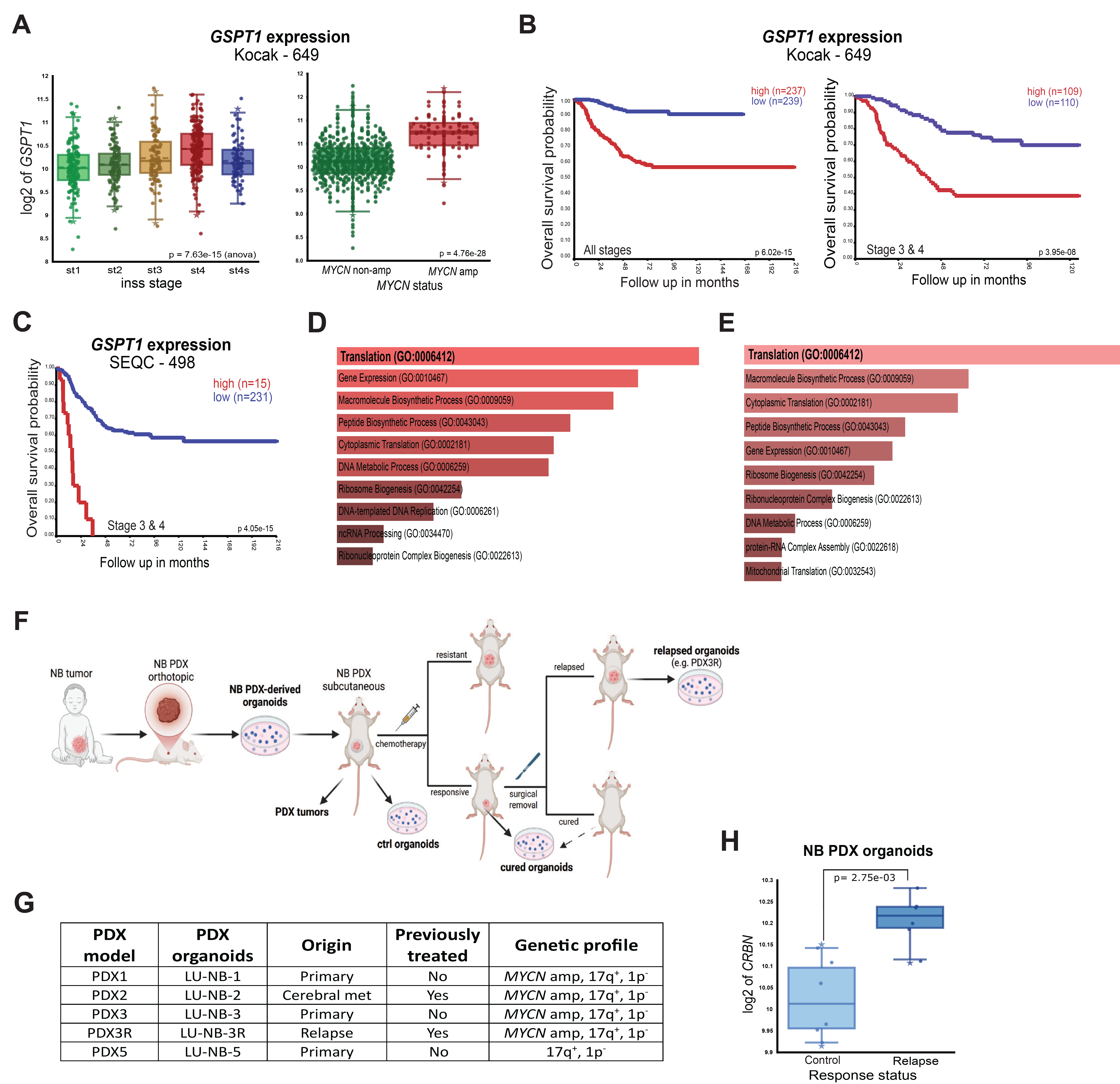


**Supplementary Figure S1. Expression of *GSPT1* and *CRBN* in NB.**

1. *GSPT1* mRNA expression in the Kocak (n=649) NB patient dataset. From left to right: box plots comparing mRNA expression based on INSS stage and *MYCN* amplification status. One-way ANOVA was used for statistical analysis, asterisks indicate outliers; **B**) Survival of patients with NB expressing high and low gene expression of *GSPT1* in the Kocak dataset, presented using Kaplan-Meier survival curves. Full patient set (left) and stage 3 and 4 patients (right) (red, high *GSPT1*, blue, low *GSPT1,* log-rank test, median used as cut-point for analysis); **C**) Survival of patients with NB expressing high and low gene expression of *GSPT1*, stage 3 and 4 patients in the SEQC (n=498) dataset (red, high *GSPT1* expression, blue- low *GSPT1,* log-rank test, scan used as cut-point for analysis); **D**) Gene Ontology analysis of the 1000 most differentially expressed genes between HR and low-risk NB patient tumors in the SEQC (n=498) dataset (R2 platform). Two-tailed *t*-test, *p* < 0.01 was used for analysis; **E**) Gene Ontology analysis of the 1000 most differentially expressed genes between *MYCN*-amplified and *MYCN* non-amplified NB patient tumors in SEQC (n=498) dataset (R2 platform). Two-tailed *t*-test, *p* < 0.01 was used for analysis; **F)** Schematic overview of the experimental workflow used for generation of NB PDX tumors and PDX-derived organoids (21); **G**) Summary of molecular features of the NB PDX models employed in the study; **H**) Comparison of *CRBN* mRNA expression between PDX3-derived organoids established from untreated PDX3 tumors (Control) and PDX3 tumors that relapsed after chemotherapy (Relapse) (21). T-test with Welch correction was used for statistical analysis.

**Supplementary Fig S2**


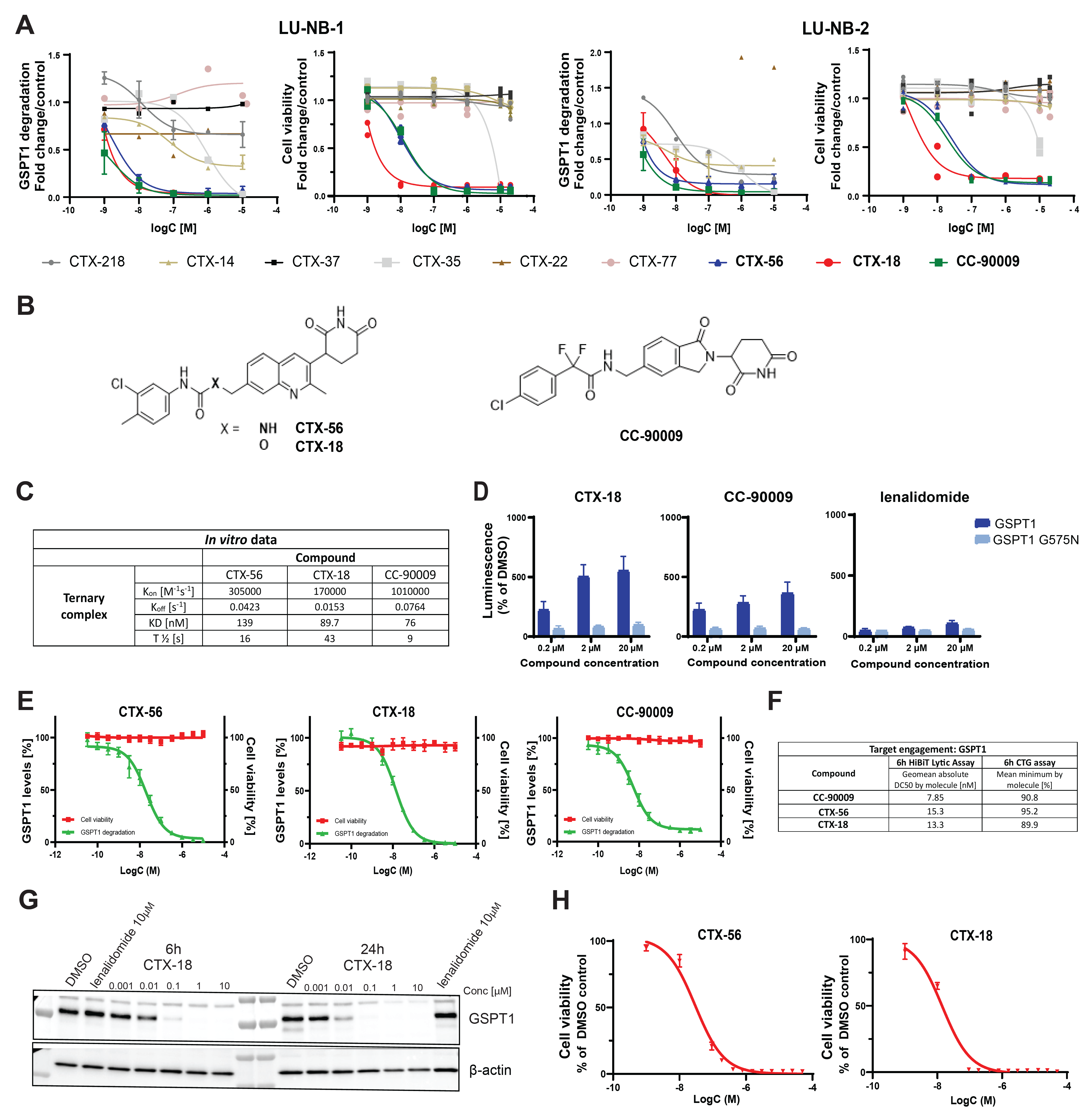


**Supplementary Figure S2. Characterization of GSPT1-degrading molecular glues.**

**A**) Drug screen of nine molecular glues (MGs) assessing their effectiveness in GSPT1 degradation (WB analysis after dose-increasing treatment for 24 h; left) and cell viability (72 h, right) in LU-NB-1 and LU-NB-2 organoids. MGs selected for further analysis are bolded; **B**) Chemical structures of the reference compound CC-90009 (right) and MGs CTX-56 and CTX-18 (left) assessed in the study; **C**) Summary of the biophysical characteristics of MGs in terms of ternary complex formation. Presented values are SPR-derived kinetic data for GSPT1 binding to CRBN-MG complexes; **D**) Dose-response binding of CTX-18 to WT GSPT1 and GSPT1 with G575N mutation in HEK293 cells. The reference compound CC-90009 was used as a positive control and CRBN-recruiting MG (lenalidomide) as negative control; **E**) Cell viability (red) and in-cell GSPT1 degradation (green) in HEK293 cells with HiBiT-tagged GSPT1 after 6 h treatment with CTX-56, CTX-18 and CC-90009. The results are presented as a mean of 2 (GSPT1 degradation) or 3 (viability) biological replicates; **F**) Summary table presenting the effects of tested MGs on viability (CTG assay) and in-cell GSPT1 degradation (DC_50_) in HEK293 cells with HiBit-tagged GSPT1; **G**) Western blot analysis of GSPT1 degradation in NB Kelly cells after treatment with CTX-18 for 6 and 24 h. Lenalidomide was used as negative control; **H**) Cell viability of NB Kelly cells following treatment with CTX-56 and CTX-18 for 72 h. The results are presented as a mean of four (CTX-56) or two (CTX-18) biological replicates.

**Supplementary Fig S3**


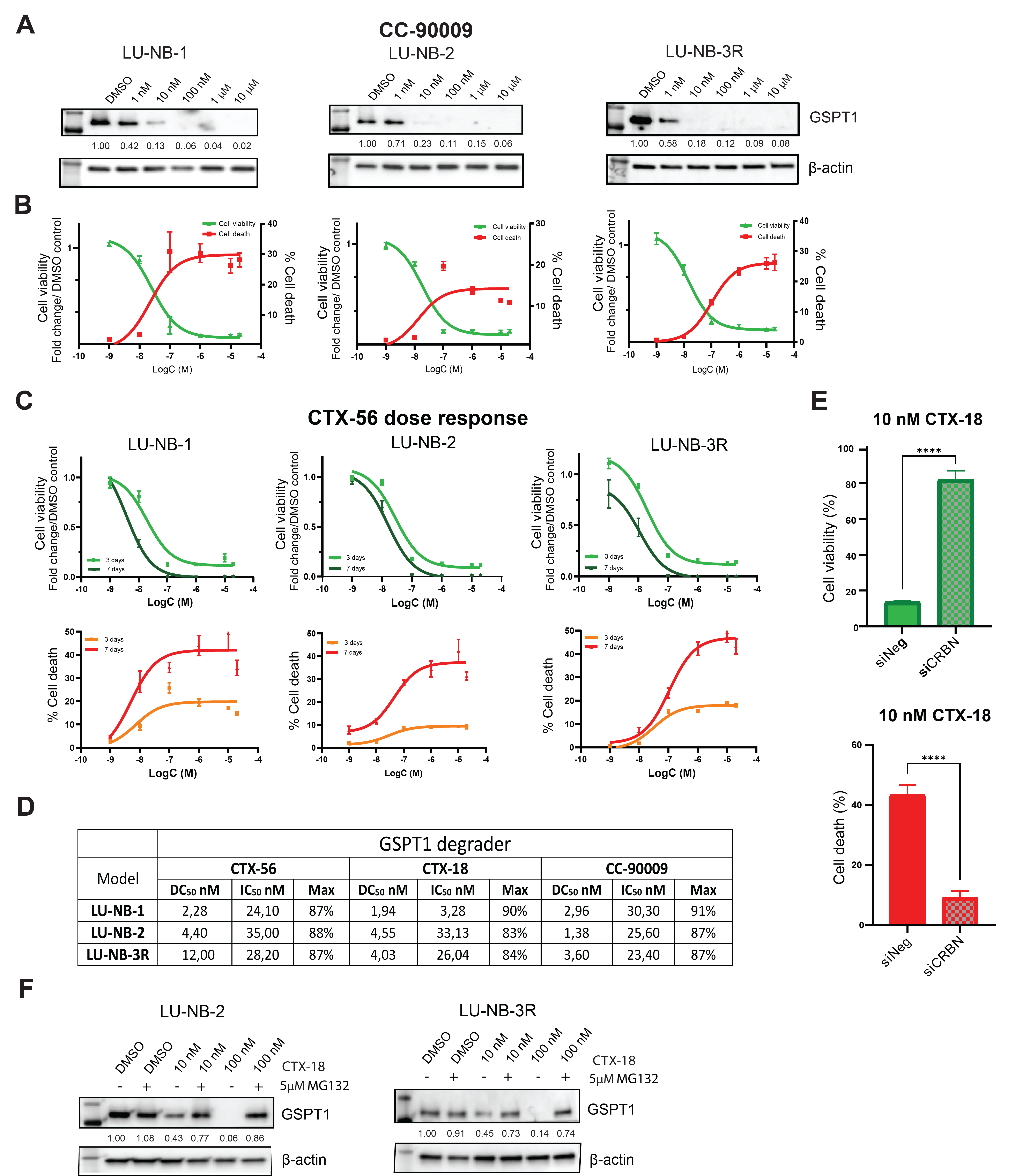


**Supplementary Figure S3. GSPT1-degradation in NB organoids.**

**A**) Western blot analysis of GSPT1 levels in NB organoids after treatment with the reference compound CC-90009 for 24 h. Quantification shows the fold change expression compared with DMSO control presented as the mean of three biological replicates; **B**) Cell viability (green) and cell death (red) in NB organoids after treatment with the reference compound CC-90009 for 72 h. 3 biological replicates (x3 technical replicates) were performed for each model; **C**) Cell viability (upper row) and cell death (lower row) in NB organoids 3 days and 7 days after CTX-56 treatment. NB organoids were re-treated at day 3. 3 biological replicates (x3 technical replicates) were performed; **D**) Summary of the effects of MGs on GSPT1 degradation (DC_50_) and viability (IC_50_ and Maximal decrease in viability) of NB organoids. The results show the mean of three biological replicates; **E**) Cell death and cell viability following treatment with 10 nM CTX-18 in LU-NB-1 organoids with/without CRBN knockdown. Treatment was performed for 48 hours. Data represent 3 biological replicates. Two-tailed *t*-test with Welch’s correction was used for analysis; **F**) Western blot analysis of GSPT1 levels following co-treatment with CTX-18 and proteasome inhibitor MG132 in NB organoids. Cells were pre-treated with 5 µM MG132 for 1 h followed by 6 h co-treatment with CTX-18 at 10 nM and 100 nM. Quantification shows fold change in expression compared with DMSO control presented as the mean of 3 biological replicates**.**

**Supplementary Fig S4**


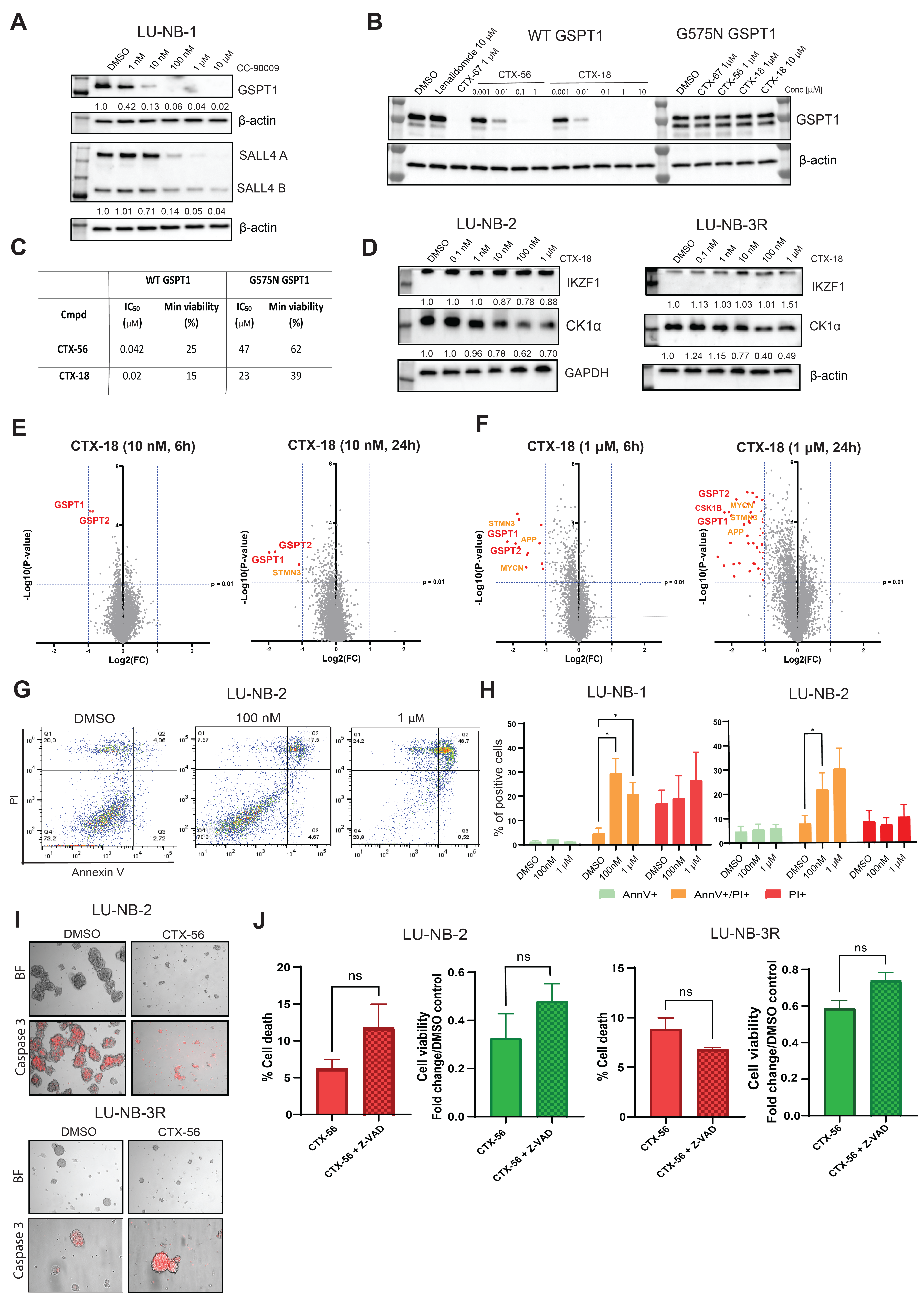


**Supplementary Figure S4. Specific GSPT1-degradation in NB organoids.**

**A**) Western blot analysis of GSPT1 and SALL4 following 24 h treatment of NB organoids with CC-90009. Quantification shows the fold change expression of SALL4A compared with DMSO control presented as the mean of three biological replicates; **B**) Western blot analysis comparing GSPT1 degradation by CTX-56 and CTX-18 in Hep3B cells expressing WT GSPT1 and G575N-mutated GSPT1. Lenalidomide (CRBN-recruiting MG with no specificity to GSPT1) was used as a negative control. CTX-67 was used as a positive control; **C**) Summary of the effects of CTX-56 and CTX-18 treatment on viability (IC_50_ and min viability) of Hep3B cells expressing WT GSPT1 or G575N-mutated GSPT1; **D**) Western blot analysis of GSPT1 and the common CRBN neosubstrates IKFZ1 and CK1α following treatment of LU-NB-2 and LU-NB-3R with CTX-18 for 24 h. Quantification shows fold change expression compared with DMSO control presented as the mean of three biological replicates; **E**) Proteomic analysis showing downregulated proteins following treatment of NB LU-NB-2 organoids with 10 nM CTX-18 for 6 h (left) or 24 h (right). ~6400 proteins were detected. Downregulated proteins are marked in red. A p-value of 0.01 and fold change of 2 was considered significant. The data shows the results from 4 biological replicates; **F**) Proteomic analysis showing downregulated proteins following treatment of NB LU-NB-2 organoids with 1 µM CTX-18 for 6 h (left) or 24 h (right). ~6400 proteins were detected. Downregulated proteins are marked in red. Downregulated NB-related proteins are marked in orange. A p-value of 0.01 and fold change of 2 was considered significant. The data shows the results from 3 biological replicates; **G**) Flow cytometry analysis of Annexin V/PI positive LU-NB-2 cells 48 h after treatment with CTX-56 (100 nM, 1 µM); **H)** Statistical analysis of the changes in proportion of Annexin V/PI positive cells after treatment of LU-NB-1 (n=3) and LU-NB-2 (n=4) cells with CTK-56; **I**) Morphological changes (brightfield imaging, upper panel) and activity of caspase 3 (brightfield + fluorescence, lower panel) in LU-NB-2 and LU-NB-3R organoids 48 h after treatment with 1 µM CTX-56. 50 µm (upper panel) and 100 µm (lower panel) scale was used; **J**) Cell death and cell viability of LU-NB-2 and LU-NB-3R organoids after co-treatment with 100 nM CTX-56 and the pan-caspase inhibitor Z-VAD, compared with CTX-56 treatment alone. Organoids were pre-treated with Z-VAD for 4 h, followed by MG treatment for 48 hours. 3 biological replicates were performed. *t*-test with Welch’s correction was used for statistical analysis.

**Supplementary Fig S5**


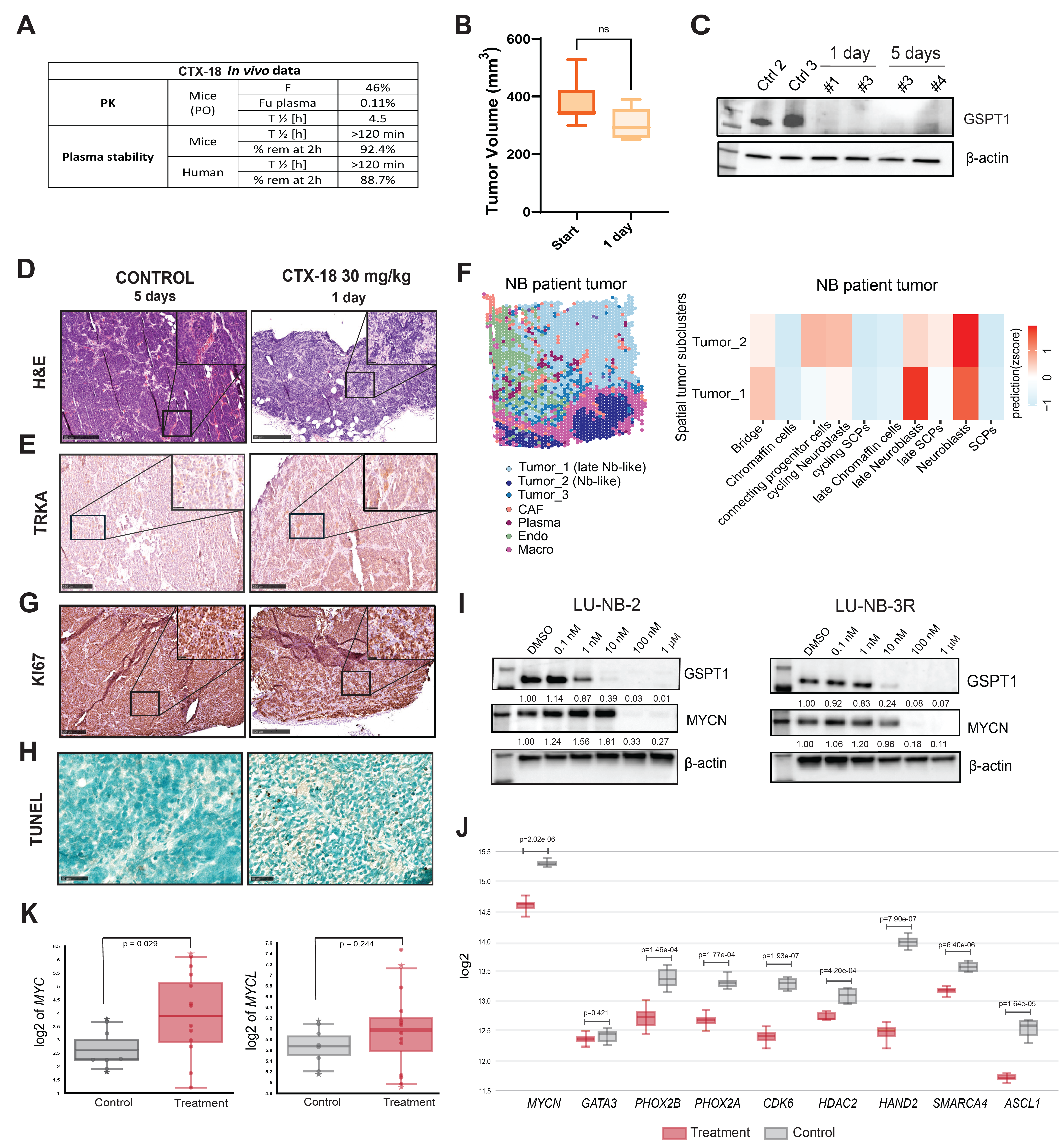


**Supplementary Figure S5. GSPT1 degradation induces NB differentiation *in vivo.***

**A)** Summary of *in vivo* PK data for CTX-18 administered by oral gavage to CD-1 mice; **B**) Tumor volume of NB PDX1 tumors following 1 day of treatment with CTX-18 (30 mg/kg) or vehicle (n=6 in each group), *t*-test with Welch’s correction was used for statistical analysis; **C**) Western blot analysis of MYCN protein levels in PDX1 tumors resected from mice treated with vehicle (Ctrl2, Ctrl3) or CTX-18 (30 mg/kg) for 1 day (1, 3) or 5 days (3, 4); **D**) H&E staining of control and CTX-18-treated PDX1 tumors after 1 day of treatment (scale bars: 500 µm and 50 µm); **E**) Representative IHC staining of TrkA in control and CTX-18-treated PDX1 tumors after 1 day of treatment (scale bars: 250 µm and 50 µm); **F**) Spatial transcriptomics of a *MYCN*-amplified NB patient tumor (27). NB tumor cell clusters were characterized according to the Jansky dataset (right); **G**) Representative IHC staining of KI67 in control and CTX-18-treated PDX1 tumors after 1 day of treatment (scale bar: 500 µm and 50 µm); **H**) Representative TUNEL staining of the control and CTX-18-treated PDX1 tumors after 1 day of treatment (scale bar: 50 µm); **I**) Western blot analysis of GSPT1 and MYCN protein expression in LU-NB-2 and LU-NB-3R organoids following treatment with CTX-18 for 24 h. The quantification is a mean of 3 biological replicates presented as fold-change levels compared with DMSO; **J**) mRNA expression levels of *MYCN* and selected *MYCN*-associated core regulatory circuitry genes in PDX1 tumors after treatment with 1 dose of CTX-18 *in vivo.* Statistical significance was assessed using a two-tailed *t*-test; **K**) mRNA expression levels of *MYC* and *MYCL* in PDX1 tumors after treatment with CTX-18 (30 mg/kg), *t*-test with Welch’s correction was used for statistical analysis.

**Supplementary Fig S6**


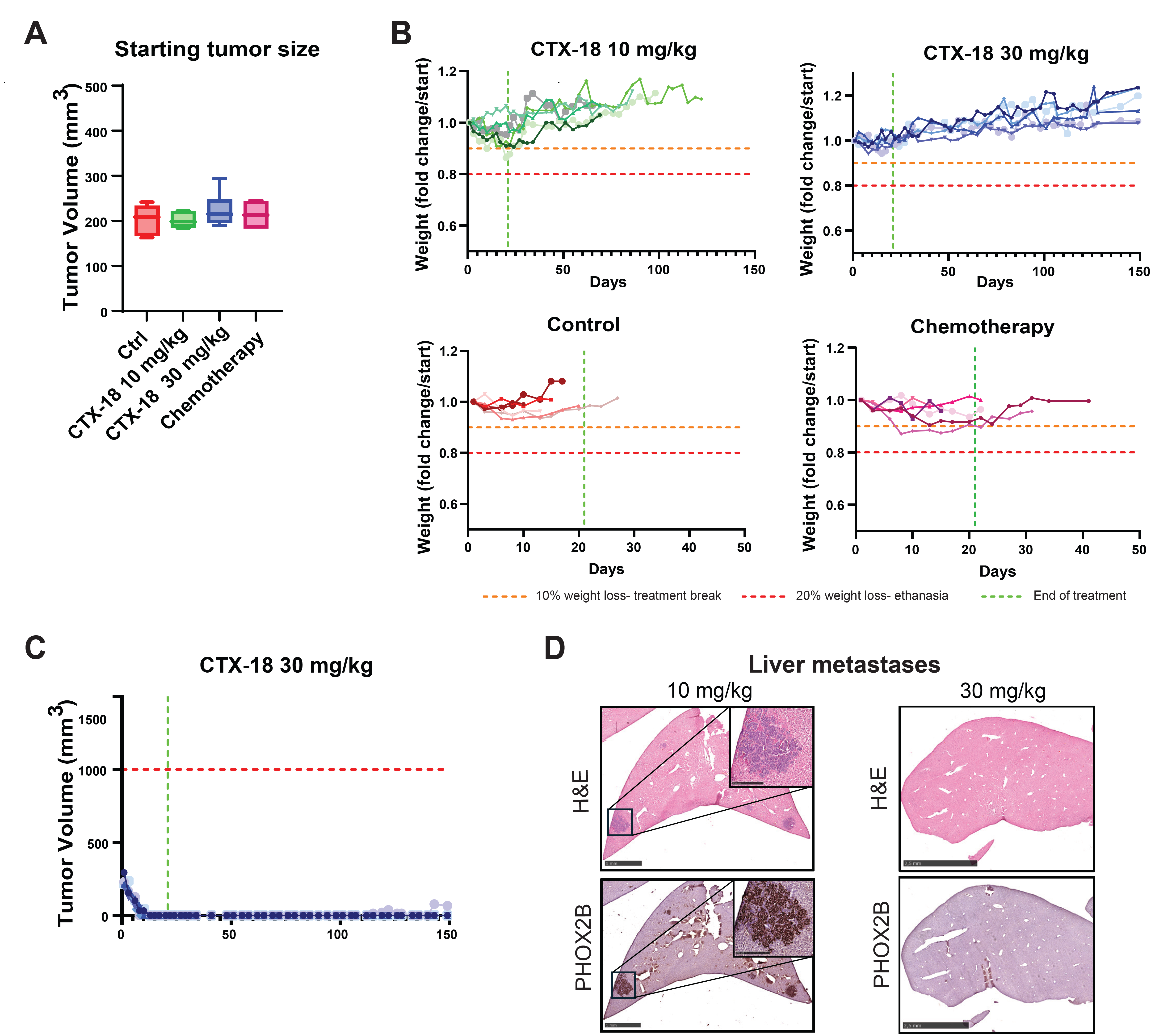


**Supplementary Figure S6. CTX-18 treatment in PDX1 NSG mouse model.**

**A**) Comparison of PDX1 starting tumor volumes across the treatment groups (n=6 mice in each group); **B**) Fold change in body weight of mice treated with vehicle control, chemotherapy, or CTX-18 (10 mg/kg and 30 mg/kg). Weights were normalized to the baseline measurement at treatment start. The green dashed line indicates end of treatment, the orange line marks weight drop below 90% of the initial weight (treatment break), the red line marks weight drop below 80% of the initial weight (euthanasia criteria); **C**) Tumor volume growth curves for individual mice treated with 30 mg/kg CTX-18 over 150 days. The green line indicates end of treatment (21 days), the red line indicates the maximal tumor volume (euthanasia criteria); **D**) Representative H&E and PHOX2B IHC staining of liver sections from PDX1-bearing mice treated with 10 mg/kg CTX-18 (left) or 30 mg/kg CTX-18 (right). Liver tissues were collected at the end point of the experiment (scale bars: 1 mm and 250 µm).
