# Supplementary material for: GSPT1-specific protein degradation is effective in preclinical models of chemoresistant MYCN-amplified neuroblastoma
Source: J Exp Clin Cancer Res. 2026 Feb 6;45:58. doi: 10.1186/s13046-026-03647-0 (PMC12918055; doi:10.1186/s13046-026-03647-0)
Supplement: Supplementary file 8 — Supplementary Material 8: Table S1_proteomics. [file 13046_2026_3647_MOESM8_ESM.docx]

|  |  |  |  |  | **LU-NB-1** | | **LU-NB-2** | |
| --- | --- | --- | --- | --- | --- | --- | --- | --- |
| **#** | **Protein name** | | **Uniprot ID** | **G-loop presence [clashscore]** | **Log2(FC)** | **-Log10 (P-value)** | **Log2(FC)** | **-Log10 (P-value)** |
| 1 | GSPT1 | Eukaryotic peptide chain release factor GTP-binding subunit ERF3A | P15170 | G-loop present [10.29] | -2.12 | 6.78 | -2.17 | 3.37 |
| 2 | GSPT2 | Eukaryotic peptide chain release factor GTP-binding subunit ERF3B | Q8IYD1 | G-loop present [23.68] | -1.50 | 5.28 | -1.92 | 3.31 |
| 3 | STMN3 | Stathmin-3 | Q9NZ72 | not found | -1.90 | 6.57 | -1.82 | 4.12 |
| 4 | MYCN | N-myc proto-oncogene protein | P04198 | not found | -1.44 | 7.62 | -1.59 | 2.51 |
| 5 | APP | Amyloid-beta precursor protein | P05067 | G-loop present [11.11] | -1.24 | 5.90 | -1.81 | 3.18 |
|  |  |  |  |  | -1.26 | 4.57 |  |  |
| 6 | CHGA | Chromogranin-A | P10645 | not found | -1.57 | 5.94 | -1.04 | 2.98 |
| 7 | SYT11 | Synaptotagmin-11 | Q9BT88 | not found | -1.08 | 4.32 | -1.11 | 2.63 |
| 8 | GOLM1 | Golgi membrane protein 1 | Q8NBJ4 | not found | -1.10 | 6.51 | -0.69 | 2.46 |
| 9 | MORF4L1 | Mortality factor 4-like protein 1 | Q9UBU8 | not found | -1.02 | 5.15 | -0.64 | 2.99 |
| 10 | MORF4L2 | Mortality factor 4-like protein 2 | Q15014 | not found | -1.08 | 4.34 | -0.70 | 3.33 |
| 11 | DLK1 | Protein delta homolog 1 | P80370 | not found | -1.54 | 8.51 | not detected | |
| 12 | RNF138 | E3 ubiquitin-protein ligase RNF138 | Q8WVD3 | G-loop present [21.5] | -1.02 | 7.38 | not detected | |
| 13 | ITM2C | Integral membrane protein 2C | Q9NQX7 | G-loop present [82.83] | -1.62 | 5.21 | not detected | |
| 14 | IGFBPL1 | Insulin-like growth factor-binding protein-like 1 | Q8WX77 | not found | -1.57 | 5.09 | not detected | |
| 15 | SYT4 | Synaptotagmin-4 | Q9H2B2 | G-loop present [1.99] | -1.52 | 4.12 | not detected | |
| 16 | ITM2B | Integral membrane protein 2B | Q9Y287 | not found | -1.32 | 4.04 | not detected | |
| 17 | PRAME | Melanoma antigen preferentially expressed in tumors | P78395 | G-loop present [0.06] | -1.20 | 3.87 | not detected | |
| 18 | FBLN1 | Fibulin-1 | P23142 | G-loop present [45.41] | -1.32 | 3.51 | not detected | |
| 19 | TNFRSF19 | Tumor necrosis factor receptor superfamily member 19 | Q9NS68 | not found | -1.18 | 3.09 | not detected | |
| 20 | MRFAP1 | MORF4 family-associated protein 1 | Q9Y605 | not found | -1.54 | 2.97 | not detected | |
| 21 | EHD3;EHD4 | EH domain-containing protein 3; EH domain-containing protein 4 | Q9NZN3, Q9H223 | not found | -1.10 | 2.77 | not detected | |
| 22 | MYBL2 | Myb-related protein B | P10244 | not found | -1.31 | 2.27 | not detected | |
| 23 | CUEDC2 | CUE domain-containing protein 2 | Q9H467 | not found | not detected | | -1.86 | 4.31 |
| 24 | PCDH8 | Protocadherin-8 | O95206 | G-loop present [153.39] | not detected | | -1.14 | 3.94 |
| 25 | B2M | Beta-2-microglobulin | P61769 | not found | not detected | | -1.19 | 3.32 |
| 26 | TRIB2 | Tribbles homolog 2 | Q92519 | G-loop present [11.84] | not detected | | -1.58 | 2.93 |
| 27 | CST3 | Cystatin-C | P01034 | not found | not detected | | -1.09 | 2.46 |
|  |  |  |  |  |  |  |  |  |
| **Known CRBN off-targets** | | | | | | | | |
| **#** | **Protein name** | | **Uniprot ID** | **G-loop presence [clashscore]** | **Log2(FC)** | **-Log10(P-value)** | **Log2(FC)** | **-Log10(P-value)** |
| 1 | SALL4 | Sal-like protein 4 | Q9UJQ4 | G-loop present [2.27] | -0.03 | 0.30 | not detected | |
| 2 | CSNK1A1 (CK1a) | Casein kinase I isoform alpha | P48729 | G-loop present [1.90] | -0.28 | 5.11 | -0.19 | 1.01 |
| 3 | IKZF1 | DNA-binding protein Ikaros | Q13422 | G-loop present [0.16] | -0.04 | 0.23 | not detected | |

**Table S1_Proteomics. Proteins that were reduced by >2-fold during treatment with 1 µM CTX-0107918 compound for 6 hours in at least one of the organoid models, and proteomic data for selected known CRBN neosubstrates.**Column 4 reports the bioinformatic assessment for G-loop presence in the analyzed protein. For a sequence that can act as a G-loop, the clashscore parameter is provided. If there is more than one potential G-loop, the clashscore value with the lowest value is provided. Color code: green - present G-loop motif with clashscore lower than the value observed for GSPT2; orange - present G-loop with clashscore higher than value observed for GSPT2; red - no G-loop motif identified; grey - change in protein abundance lower than 2-fold.
